# Evolutionary Diversification of Plant Shikimate Kinase Gene Duplicates

**DOI:** 10.1371/journal.pgen.1000292

**Published:** 2008-12-05

**Authors:** Geoffrey Fucile, Shannon Falconer, Dinesh Christendat

**Affiliations:** Department of Cell and Systems Biology, University of Toronto, Canada; The Univerity of British Columbia, Canada

## Abstract

Shikimate kinase (SK; EC 2.7.1.71) catalyzes the fifth reaction of the shikimate pathway, which directs carbon from the central metabolism pool to a broad range of secondary metabolites involved in plant development, growth, and stress responses. In this study, we demonstrate the role of plant *SK* gene duplicate evolution in the diversification of metabolic regulation and the acquisition of novel and physiologically essential function. Phylogenetic analysis of plant SK homologs resolves an orthologous cluster of plant SKs and two functionally distinct orthologous clusters. These previously undescribed genes, *shikimate kinase-like 1* (*SKL1*) and -*2* (*SKL2*), do not encode SK activity, are present in all major plant lineages, and apparently evolved under positive selection following *SK* gene duplication over 400 MYA. This is supported by functional assays using recombinant SK, SKL1, and SKL2 from *Arabidopsis thaliana* (At) and evolutionary analyses of the diversification of SK-catalytic and -substrate binding sites based on theoretical structure models. At*SKL1* mutants yield albino and novel variegated phenotypes, which indicate *SKL1* is required for chloroplast biogenesis. Extant *SKL2* sequences show a strong genetic signature of positive selection, which is enriched in a protein–protein interaction module not found in other *SK* homologs. We also report the first kinetic characterization of plant SKs and show that gene expression diversification among the At*SK* inparalogs is correlated with developmental processes and stress responses. This study examines the functional diversification of ancient and recent plant *SK* gene duplicates and highlights the utility of SKs as scaffolds for functional innovation.

## Author Summary

Gene duplicates provide an opportunity for functional innovation by buffering their ancestral function. Mutations or genomic rearrangements altering when and where the duplicates are expressed, or the structure/function of the products encoded by the genes, can provide a selective advantage to the organism and are subsequently retained. In this study, we demonstrate that duplicates of genes encoding the metabolic enzyme shikimate kinase (SK) in plants have evolved to acquire novel gene product functions and novel gene expression patterns. We introduce two ancient genes, *SKL1* and *SKL2*, present in all higher plant groups that were previously overlooked due to their overall similarity to the ancestral SKs from which they originated. *SKL1* mutants in the model plant Arabidopsis indicate this gene is required for chloroplast biogenesis. We show that *SKL2* acquired a protein–protein interaction domain that is evolving under positive selection. We also show that SK duplicates that retained their ancestral enzyme function have acquired new expression patterns correlated with developmental processes and stress responses. These findings demonstrate that plant SK evolution has played an important role in both the acquisition of novel gene function as well as the diversification of metabolic regulation.

## Introduction

The shikimate pathway functions at a critical interface between primary and secondary metabolism by channeling carbon from glycolysis and the pentose phosphate pathway towards the synthesis of a broad range of physiologically important aromatic compounds [1]. In plants these include the aromatic amino acids, phenylpropanoids, lignins, hormones, pigments, phytoalexins, alkaloids, UV protectants, and electron carriers [2]. Metabolites of the main trunk of the shikimate pathway are also considered branch point substrates for other secondary metabolic pathways [1]. Shikimate kinase (SK; EC 2.7.1.71) catalyzes the fifth reaction of the shikimate pathway with the phosphorylation of shikimate to shikimate-3-phosphate using ATP. It has been suggested that plant SKs act as regulatory points for the shikimate pathway, facilitating metabolic flux towards specific secondary metabolite pools [3]. This is supported by observations of rapid induction of plant SK transcripts by fungal elicitors [4], the significant sensitivity of plant SK activity to cellular ATP energy charge [5], and the differential expression of the three rice SK genes during specific developmental stages and biotic stress response [6].

Towards understanding the role of plant SKs in metabolic regulation we assessed the functional significance of plant SK gene duplicate evolution. Plant species typically exhibit an increased rate of gene duplicate retention compared to other organisms [7]–[8], which suggests duplicate genes perform a prominent role in many aspects of plant physiology. The evolution of gene duplicates has been proposed as a central mechanism for the diversification of compounds produced by plant secondary metabolism and the regulation of these metabolic pathways [9]–[12]. In addition to providing genetic robustness against deleterious mutations through functional redundancy, current theory posits the retention of duplicate plant loci by positive selection following advantageous sub- or neofunctionalization of gene expression patterns or gene product function [13]. Subfunctionalization, also referred to as the duplication-degeneration-complementation model [14], involves the splitting of cis-regulatory sequences in a gene's promoter or the encoded functions of the gene product among the gene duplicates. Neofunctionalization refers to the acquisition of a new function of the encoded gene product [15] or a new spatial or temporal gene expression pattern. The gain and loss of sequence elements following gene duplication can lead to important functional innovations in shikimate pathway enzymes. For example, Ding *et al.* suggest that the loss of a chloroplast transit peptide in a duplicate of the bifunctional dehydroquinate dehydratase/shikimate dehydrogenase in Tobacco may contribute to partitioning of plant shikimate pathway flux to the cytosol, or represent a novel enzyme [16].

In this study we show that the evolution of independent plant *SK* gene duplicates has led to the acquisition of novel gene function and the diversification of metabolic regulation. We use phylogenetic and biochemical approaches to resolve a functionally orthologous group of plant *SK* genes and present the first kinetic characterization of plant SKs, *Arabidopsis thaliana* (At) SK1 and SK2. The genes encoding these enzymes arose from a recent segmental duplication and subsequently underwent regulatory neofunctionalization. This gene expression variation between these inparalogs is correlated with developmental processes and stress responses. We also report two previously undescribed and functionally distinct groups of *SK* homologs, shikimate kinase-like 1 (*SKL1*) and 2 (*SKL2*), which arose following *SK* gene duplication over 400 MYA. Characterization of albino and novel variegated T-DNA insertion alleles of At*SKL1* indicate that the gene product is essential for chloroplast biogenesis. *SKL2* acquired a protein–protein interaction module which carries a strong genetic signature of positive selection within the plant *SKL2* family, suggesting the encoded protein is important for species-specific adaptive molecular evolution. The role of *SK* gene duplicate diversification in the acquisition of novel enzyme function and regulation of metabolic flux is discussed.

## Results

### Phylogenetic Reconstruction of Plant SK Homologs Defines Novel Orthologous Clusters

**Figure 1:**
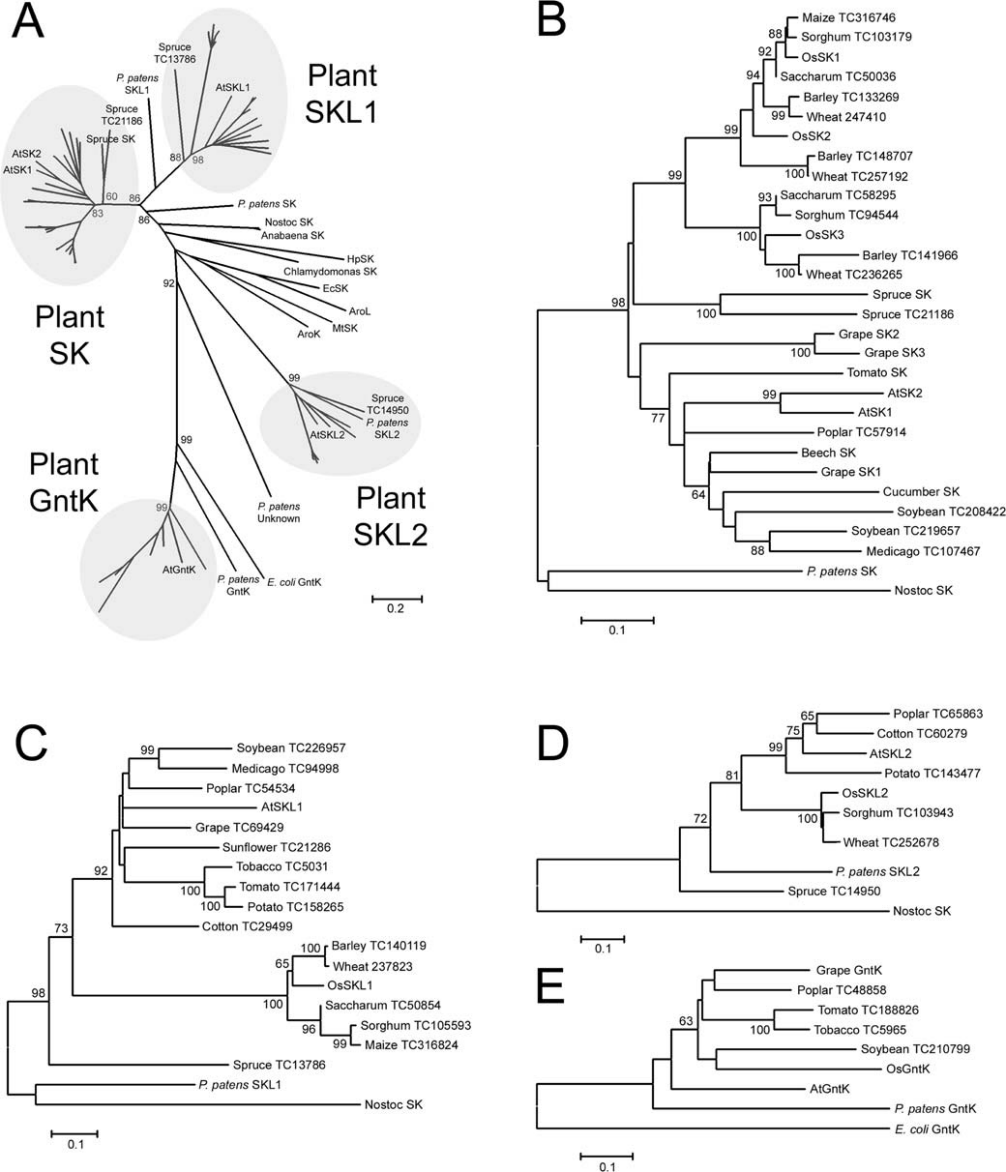
Phylogenetic relationship of plant and microbial SK homologs. A) Unrooted phylogenetic reconstruction of plant and microbial SK homologs using the neighbor-joining algorithm. Phylogenetic reconstructions of the B) plant SK family, C) plant SKL1 family, D) plant SKL2 family, and E) plant GntK family generated using the neighbor-joining method. Scores for 1000 bootstrap iterations over 60 are indicated. Ec; *Erwinia crysanthemi*, Hp; *Helicobacter pylori*, Mt; *Mycobacterium tuberculosis*, AroK; *Escherichia coli* SKI, AroL; *Escherichia coli* SKII. Accession numbers for GenBank sequences are listed in Table S1. TIGR EST assemblies are identified by Tentative Consensus (TC) numbers.

To assess the degree of *SK* gene duplicate retention in plants we retrieved plant SK homologs by sequence similarity searches using the functionally characterized tomato (*Lycopersicon esculentum*) and rice (*Oryza sativa*) SK amino acid sequences. The retrieved collection of protein sequences was used to build a boot-strapped neighbor-joining phylogenetic reconstruction (Figure 1A). The out-groups consist of plant gluconate kinase (GntK, EC: 2.7.1.12) sequences, which are closely related to SKs in the nucleoside monophosphate kinase (NMPK) family but functionally distinct (Figure S1) [17]–[18], and microbial SKs with a BLASTp significance threshold of E<10e-30 against plant SK homologs. The plant SK (Figure 1B), SKL1 (Figure 1C), and SKL2 (Figure 1D) clades of this tree have sequences from each of the major land plant lineages of monocots, dicots, and conifers. There is a clear SKL2 ortholog in the moss *Physcomitrella patens* genome. There also appears to be SK and SKL1 orthologs in *P. patens*, however they do not resolve distinctly into the plant clades.

Monocot and dicot sequences are split into sub-groups and are derived from moss and spruce sequences in these clades which indicate they are separate orthologous clusters. The functionally characterized tomato and rice SKs are in the plant SK clade, which has two or more SK paralogs for 9 of the 15 species represented in this clade (Figure 1B). There are groups of SK paralogs in the monocot lineage whereas dicot SK paralogs are species-specific (inparalogs). The SKL1 clade is similarly distant (amino acid substitutions per site) to SKs from plants (1.22+/−0.07) and microbes (1.58+/−0.10), whereas the SKL2 clade is more distantly related to the plant SKL1 (2.00+/−0.12) and the plant SK clades (2.12+/−0.14) (Figure 1A). Unlike the plant SKs, none of the plant species investigated carries more than one SKL1 (Figure 1C) or SKL2 homolog (Figure 1D).

**Figure 2:**
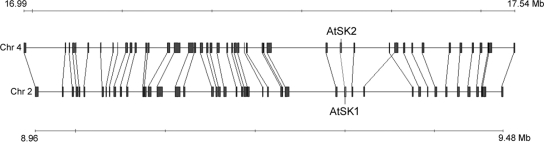
Duplication origin of AtSK1 and AtSK2. AtSK1 and AtSK2 reside in sister duplicated blocks in chromosomes 4 and 2 of *Arabidopsis thaliana*, respectively. Image modified from: http://wolfe.gen.tcd.ie/cgi-bin/athal/dup. Chr = chromosome, Mb = megabases.

The Arabidopsis genome has two *SK* homologs (*AtSK1*; At2g21940, *AtSK2*; At4g39540), and one *SKL1* (*AtSKL1*; At3g26900) and *SKL2* homolog (*AtSKL2*; At2g35500). Pair-wise amino acid percent sequence identities among these homologs are presented in Table 1. AtSK1 and AtSK2 both match the Pfam SK Markov model (PF01202) [19] with a probability of E = 10e-37. AtSKL1 matches this Pfam model with a probability of E = 10e-23 and is 35% identical at the amino acid level with AtSK1 and AtSK2. AtSKL2 matches the Pfam SK model with E = 3e-5 and is ∼22% identical to the other Arabidopsis SK homologs. AtSK1 and AtSK2 are inparalogs which arose following a recent (20–60 MYA) segmental duplication of chromosome 2 to chromosome 4 (Figure 2) [20]. AtSKL1 and AtSKL2 also reside in recently duplicated blocks. However, there are no retained duplicates in the respective sister regions of these loci.

**Table 1:** Global pairwise amino acid alignment percent identities among Arabidopsis SK homologs generated using EMBOSS with the BLOSUM30 matrix.

	AtSKL1	AtSKL2	AtSK2	AtSK1	AtGntK
AtSKL1	100				
AtSKL2	22	100			
AtSK2	33	23	100		
AtSK1	35	22	82	100	
AtGntK	21	15	18	19	100

Protein sequences compared exclude putative N-terminal transit peptide.

### Arabidopsis *SK* Inparalogs Are Differentially Expressed in Development and Stress Responses

**Figure 3:**
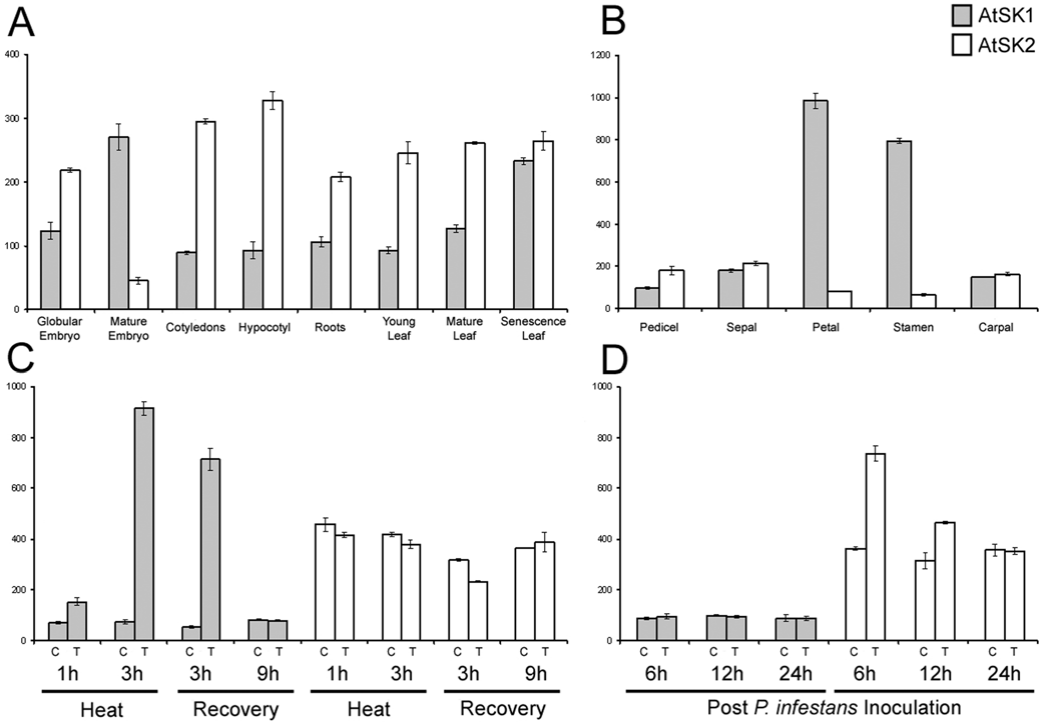
Gene expression profiles of AtSK1 and AtSK2. Transcript expression profiles of AtSK1 (grey bars) and AtSK2 (white bars) are represented as normalized mean fluorescent units of biological replicates. Average background levels (ABL) were calculated for each experiment as mean background signal values with standard deviations. A) Specific tissues during stages of development from embryogenesis to senescence (ABL = 42.9±4.6), B) specific floral organs (ABL = 49.1±6.7), C) heat stress response in shoots (ABL = 47.3±1.9), D) whole plants post-inoculation with *Phytophthora infestans* spores (ABL = 107.7±20.7). Control (C) and treatment (T) samples are indicated for C) and D). Gene expression data were retrieved from the publicly available AtGenExpress dataset [21].

We compared gene expression profiles from the AtGenExpress (ATGE) Arabidopsis Expression Atlas [21] to further characterize the similar Arabidopsis *SK* homologs, and phylogenetically distinct *SKL1* and *SKL2* clades. These transcript microarray data are represented as normalized mean fluorescent units of samples measured in biological triplicates on the Affymetrix platform. At*SK1* and At*SK2* expression patterns differ during the life cycle of Arabidopsis (Figure 3A). At*SK2* is predominantly expressed early in embryogenesis and in vegetative tissues throughout development. Conversely, At*SK1* is expressed near or below background levels in vegetative tissues and is only expressed at higher levels in mature embryos and senescing leaves. Petals and stamens also have a considerable increase in At*SK1* expression, approximately 10-fold higher than its median signal across the ATGE data set (Figure 3B). At*SK1* and At*SK2* also appear to be differentially expressed under conditions of biotic and abiotic stress. At*SK1* is highly induced during heat stress and recovery (Figure 3C) whereas At*SK2* is induced approximately 2-fold following inoculation with spores of *Phytophthora infestans* (Figure 3D). At*SKL1* and At*SKL2* share similar and relatively static expression profiles. These transcripts are only detected at significant levels in green tissues. Neither genes are significantly induced or repressed under any of the conditions tested in the TAIR microarray data sets (data not shown).

### Arabidopsis SK1 and SK2 Are Shikimate Kinases

The Arabidopsis orthologs from each of the plant SK, SKL1, and SKL2 families were recombinantly expressed and purified from soluble fractions using affinity chromatography for functional analysis. The constructs were prepared without predicted N-terminal transit peptides (denoted as Δ followed by the number of residues truncated from the N-terminus) as determined by ChloroP and secondary structure prediction (Table S2). SK activity was assayed for these recombinant proteins spectrophotometrically [22]. The AtSK1Δ55 and AtSK2Δ55 recombinants are enzymatically active SKs, with kinetic parameters (Table 2) consistent with those previously reported for *Erwinia chrysanthemi* SK (K_m *shikimate*_ = 310 µM, K_m *ATP*_ = 620 µM) [22]. However, neither AtSKL1Δ64 nor AtSKL2Δ60 were shown to phosphorylate shikimate *in vitro* (Table 2). Two other constructs of AtSKL1 were tested (AtSKL1Δ58 and AtSKL1Δ61) for reproducibility among constructs in addition to a pH range of 5.0–9.5 and the addition of zinc, manganese, calcium, and nickel divalent cations as magnesium substitutes (data not shown). Since reducing agents have been shown to enhance the activity of shikimate pathway enzymes [23], the shikimate phosphorylation assays were thus repeated with 200 uM DTT for each of the recombinant constructs. Whereas AtSK1 and AtSK2 catalyze shikimate phosphorylation, none of the recombinant SKL1 or SKL2 constructs catalyzed the SK reaction in any of the conditions tested.

**Table 2:** Saturation kinetics for SK activity coupled to NADH oxidation.

Enzyme	Substrate	K_m_ (uM)	K_cat_ (s^−1^)
AtSK2Δ55	Shikimate	422.15±31.16	136.06±4.97
	ATP	246.7±22.56	148.57±5.04
AtSK1Δ55	Shikimate	648.32±23.45	148.45±2.32
	ATP	218.02±12.27	162.43±3.01
AtSKL1Δ58	Shikimate	-	na
	ATP	-	na
AtSKL2Δ60	Shikimate	-	na
	ATP	-	na

na = no activity.

### SK Catalytic and Shikimate Binding Sites Are not Conserved in SKL1 and SKL2

Conservation of amino acid sites required for SK substrate binding and catalysis among the AtSK homologs was assessed based on microbial SK crystal structure data. The *Helicobacter pylori* (Hp) SK was co-crystallized with shikimate [24], the *Mycobacterium tuberculosis* (Mt) SK with shikimate and a non-hydrolysable ATP analog [25] and with shikimate and ADP (PDB:2IYQ), and the *Erwinia chrysanthemi* (Ec) SK with ADP [22]. These studies identified the following SK substrate binding and catalytic domains: 1) the P-Loop (Walker A) motif, GxxGxxK[S/T], which is required for stabilizing β- and γ-phosphates of bound nucleotide [22], 2) a Walker B Motif comprised of a series of three glycines and a DxD motif which interacts with the magnesium coordinated to the ATP phosphate moieties and stabilizes the active site by interaction with the P-Loop [26], 3) the shikimate binding domain (SBD), consisting of several hydrophobic residues and one proximal and one distal arginine, and 4) the LID domain, a dynamic helical/loop region containing a highly conserved RPLL consensus sequence [26]–[27] of which the arginine is proposed to participate directly in the transfer of the ATP γ-phosphoryl group to shikimate [24].

Based on amino acid alignments with HpSK, MtSK, and EcSK (Figure S2), AtSK1 and AtSK2 show strict conservation of all key microbial SK binding and catalytic residues (Table 3). AtSKL1 sites aligning to the ATP and Mg^2+^ binding residues are largely conserved, however the proximal arginine of the SK SBD and the catalytic arginine of the LID domain are not conserved. This is similar to AtGntK, which also has an enriched conservation of ATP binding residues but lacks conservation of SK shikimate binding and catalytic residues. AtSKL2 is highly divergent from SKs, with a partially conserved Walker B motif and the majority of SK shikimate and ATP binding residues not conserved.

**Table 3:** Residues involved in binding shikimate, ATP or ATP analogs, and Mg^2+^ collated from crystal structures of *Mycobacterium tuberculosis* (MtSK [27]), *Helicobacter pylori* (HpSK [24]), and *Erwinia crysanthemi* (EcSK [22]) binary and ternary complexes.

Reference Position			Substrate	AtSKL1	AtSKL2	AtSK2	AtSK1	AtGntK
HpSK	MtSK	EcSK						
Gly8	Gly9	Gly9	ATP	*	*	*	*	*
Gly11	Gly12	Gly12	ATP			*	*	*
Ser12	Ser13	Cys13	ATP	*		*	*	
Lys14	Lys15	Lys15	ATP	*		*	*	*
Ser15	Ser16	Thr16	ATP	*		*	*	*
Ser16	Thr17	Thr17	ATP			*	*	*
Asp31	Asp32	Asp32	Mg^2+^	*	*	*	*	*
Asp33	Asp34	Asp34	Shikimate/Mg^2+^	*		*	*	*
Gly79	Gly79	Gly78	Shikimate/ATP	*		*	*	
Gly80	Gly80	Gly79	Shikimate/ATP	*	*	*	*	
Gly81	Gly81	Gly80	Shikimate/ATP	*	*	*	*	
Phe48	Phe49	-	Shikimate	*		*	*	
Arg57	Arg58	Arg58	Shikimate			*	*	
Val44	Ile45	Val45	Shikimate			*	*	
Arg116	Arg117	Arg120	Shikimate			*	*	
Arg132	Arg136	Arg139	Shikimate	*		*	*	

*: indicates conservation of the indicated residue within multiple sequence alignments including Arabidopsis SK homologs generated using MAFFT [88].

**Figure 4:**
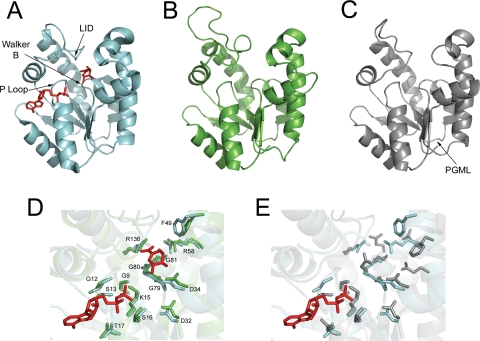
Structural models of AtSK2 and AtSKL1. A) Experimentally characterized MtSK crystal structure complexed with shikimate and ADP shown in red (PDB:1L4U), B) AtSK2 generated using MtSK as a template, C) AtSKL1 using MtSK as a template, D) superimposition of MtSK and AtSK2 active sites with ADP and shikimate shown in red, residues indicated are indexed to MtSK, E) superimposition of predicted AtSKL1 active site with MtSK with ADP shown in red. For D) and E), the catalytic arginine of the RPLL LID consensus sequence was removed from the MtSK and AtSK2 sequences for clarity.

The degree of sequence conservation between AtSK1, AtSK2 and AtSKL1 to MtSK is sufficient to build high confidence (E = 10^∧^-25, precision = 100%) *in silico* 3D models using Phyre [28] with the MtSK crystal structure (PDB:1L4U) as a template. SKL2 is not sufficiently similar to microbial SKs to build high-confidence models. The overall fold of the predicted AtSK2 (Figure 4B) and AtSKL1 (Figure 4C) models are highly similar to the experimentally determined MtSK structure. The active site architecture of the AtSK2 model includes the key SK substrate binding residues (Figure 4D). The AtSKL1 ATP binding sites are highly conserved; however the active site architecture is clearly divergent (Figure 4E).

### SKL1 and SKL2 Have Domains Distinct from Ancestral Shikimate Kinases

The domain structure of the plant SK homologs was investigated to assess functional conservation and diversification. The N-terminal regions of plant SK, SKL1, and SKL2 sequences are predicted to be chloroplast transit peptides (cTPs) (Table S2) [29], consistent with previous studies demonstrating chloroplast import of plant SKs via N-terminal cTPs [5],[30]. SKL1 has been detected in the plastid stroma of *Arabidopsis* leaf tissue by LC/MS and *Zea mays* bundle sheath cells by 2-D PAGE/MS [31]–[33]. AtSKL2 was also detected in the plastid stroma by LC/MS [32]–[33].

The highly conserved LALLRHG[I/V]S motif was evident from the SKL1 family alignment (Figure S2). PHI-BLAST analysis [34] with this motif retrieved a number of phosphoglycerate mutase (PGM; EC:5.4.2.1) sequences. Residues from this motif has been shown to bind 3-phosphoglycerate in PGM crystal structures [35], wherein His-8 forms a phosphohistidine intermediate and Arg-7 and Ser-11 form hydrogen bonds with 3-phosphoglycerate. This SKL1 phosphoglycerate mutase-like (PGML) domain is predicted to occur in a solvent exposed loop region (Figure 4C). Recombinant AtSKL1 does not have ATPase activity in the presence of glycerate or 3-phosphoglycerate.

SKL2 orthologs contain a ∼80 amino acid domain downstream of the cTP with similarity to the Pfam CS (CHORD and SGT1) domain model (PF04968) (Figure S3). SKL2 expect values for PF04968 are typically <1e-6, although the spruce SKL2 sequence scores E = 6e-16. The CS domain is a binding module for both HSP90 and CHORD domain-containing proteins [36] suggesting this SKL2 region facilitates a protein–protein interaction. Both the Physcomitrella and Spruce SKL1 and SKL2 orthologs possess the PGML and CS domains, respectively (Figure S2).

### Purifying and Positive Selection Drove Diversification of *SKL1* and *SKL2*

We predict that functionally important amino acids in the *SKL1* and *SKL2* families may be under purifying selection. The codeml module of PAML 4.0 [37] was used to identify codons that show a genetic signature consistent with this selection pressure. The likelihoods and parameter estimates for models M0 (one ratio), M3 (discrete), M1a (neutral), and M2a (selection) applied against the plant SK, SKL1, and SKL2 families aligned by codons in MEGA 4.0 are presented in Table 4. The M0 model assumes a single ratio of non-synonymous:synonymous substitution rates (ω) for all branches in the phylogeny and all sites in the gene, and was compared against the M3 model which allows for three discrete site class ω ratios that are estimated from the data. The M1a model allows only two site classes, neutral (ω = 1) and purifying selection (0<ω<1), and was compared against M2a, which allows for a third site class of positive selection (ω>1). Likelihood ratio tests (LRT) were performed for M0/M3 and M1a/M2a. From these models, purifying selection was inferred for ω<1 and positive selection was inferred for ω>1. We were unable to reject the neutral (M1a) model for both the *SK* and *SKL1* families, neither of which have any aligned residues with ω>1. Conversely, the M1a/M2a LRT indicates the plant *SKL2* family is under significant positive selection. The M0/M3 LRT similarly found significant rate heterogeneity among amino acid residues in the SKL2 family.

**Table 4:** PAML codeml module likelihood scores and parameter estimates for MAFFT nucleotide alignments of the plant *SK*, *SKL1* and *SKL2* families.

Gene Family	Model	likelihood	ts/tv	Average dN/dS	Parameter Estimates	
					Frequency	dN/dS
***SK***	M0, one-ratio	−9019.68	1.98	0.157	p = 1.000	ω = 0.157
	M3, discrete	−8809.15	2.13	0.184	p_0_ = 0.344	ω_0_ = 0.025
					p_1_ = 0.398	ω_1_ = 0.145
					p_2_ = 0.258	ω_2_ = 0.457
	M1a, neutral	−8909.16	2.30	0.311	p_0_ = 0.78848	ω_0_ = 0.12549
					p_1_ = 0.21152	ω_1_ = 1.00000
	M2a, selection	−8909.16	2.30	0.311	p_0_ = 0.78847	ω_0_ = 0.12548
					p_1_ = 0.21153	ω_1_ = 1.0000
					p_2_ = 0.00000	ω_2_ = 6.99385
***SKL1***	M0, one-ratio	−6310.96	1.89	0.189	p = 1.000	ω = 0.189
	M3, discrete	−6187.15	1.95	0.231	p_0_ = 0.252	ω_0_ = 0.022
					p_1_ = 0.591	ω_1_ = 0.178
					p_2_ = 0.157	ω_2_ = 0.761
	M1a, neutral	−6216.0363	2.02	0.281	p_0_ = 0.82812	ω_0_ = 0.13190
					p_1_ = 0.17188	ω_1_ = 1.00000
	M2a, selection	−6216.0363	2.02	0.281	p_0_ = 0.82812	ω_0_ = 0.13190
					p_1_ = 0.17181	ω_1_ = 1.00000
					p_2_ = 0.00006	ω_2_ = 1.00000
***SKL2***	M0, one-ratio	−8244.72	1.46	0.272	p = 1.000	ω = 0.272
	M3, discrete	−8087.24	1.46	0.123	p_0_ = 0.899	ω_0_ = 0.020
					p_1_ = 0.081	ω_1_ = 0.695
					p_2_ = 0.020	ω_2_ = 2.498
	M1a, neutral	−8623.98	1.31	0.087	p_0_ = 0.91628	ω_0_ = 0.00346
					p_1_ = 0.08372	ω_1_ = 1.00000
	M2a, selection	−8165.8	1.46	0.122	p_0_ = 0.92524	ω_0_ = 0.02280
					p_1_ = 0.06438	ω_1_ = 1.00000
					p_2_ = 0.01038	ω_2_ = 3.49887

	Likelihood Ratio Test	2ΔL	Df	P
***SK***	M2-M1	0	2	-
***SKL1***	M2-M1	0	2	-
***SKL2***	M2-M1	916.4	2	<0.001

Ts = transition, tv = transversion, Df = degrees of freedom, P = probability, 2ΔL = twice the difference of log likelihood values.

**Figure 5:**
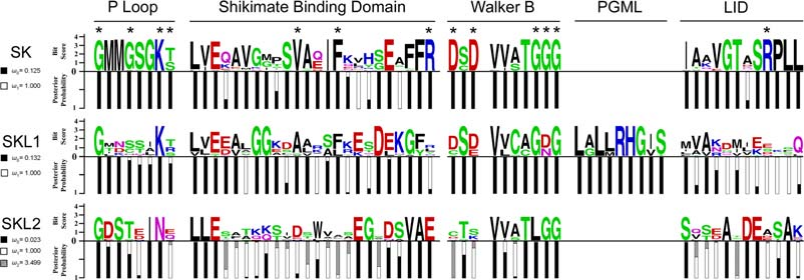
Conservation of functional domains and modes of selection acting on plant SK homologs. Stacked histograms indicate posterior probabilities of purifying (black), neutral (white), and positive selection (hatched) for sites aligning with key SK catalytic and substrate binding domains indexed to sequence logos for MAFFT amino acid alignments of the plant SK, SKL1, and SKL2 families. * indicates SK residues directly involved in substrate interactions as determined from microbial SK crystallographic structures (see Table 3). The distal arginine of the SK SBD is not shown.

The codeml module of PAML was used to calculate the posterior probability of each codon belonging to a particular site class, and then aligned to sequence logos [38] of the plant SK, SKL1, and SKL2 families to highlight modes of selection on sites within conserved domains (Figure 5). As expected, the majority of residues in SK domains within the plant SK family are under purifying selection, including all key binding and catalytic residues (87% of sites with ω<0.130). The plant SKL1 clade shows conservation of most of the ATP binding (P-loop and Walker B) domains with all sites under purifying selection in these regions. Some SKL1 sites aligning to the SK SBD are also conserved under purifying selection. However, the SKL1 site aligning to the proximal arginine of the SK SBD is not conserved and is under relaxed selective pressure (ω = 0.997). SKL1 sites that correspond to the SK LID domain region are also selectively neutral and show no conservation of the RPLL motif, which contains the catalytic arginine of plant and microbial SKs. The SKL1 PGML domain, which is not present in the plant SK or SKL2 families, is under strong purifying selection.

The SKL2 family has a dramatically different pattern of selection than the SK and SKL1 family, and shows a significant signature of positive selection for 18% of aligned sites (Table S3), including 31% of the SKL2 CS domain and sites aligning to SK substrate binding and catalytic domains. Substitutions are not saturated in the SKL2 family, as determined by a linear relationship between substitution frequencies and genetic distance and the Xia index (p<0.0001) [39] (data not shown). Substitution saturation is marginally significant for the SKL1 family (p = 0.07) and highly significant for the SK family (p<0.0001). The latter does not affect these findings, as positive selection was not detected for the SK or SKL1 families and the phylogenetic reconstructions (Figure 1) are based on amino acid translations.

### Mutations in At*SKL1* Yield Chloroplast Biogenesis Defects

**Figure 6:**
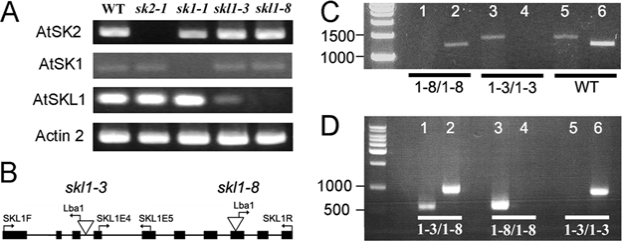
Transcript expression and genetic characterization of TDNA mutants of Arabidopsis *SK* homologs. A) Full-length coding sequence RT-PCR of AtSK2, AtSK1, and AtSKL1 from non-transgenic Arabidopsis seedlings (WT) and homozygous TDNA insertion mutants *sk2-1*, *sk1-1, skl1-8*, and *skl1-3*. Actin2 RT-PCR is included as a control for each genotype. B) Schematic representation of *skl1-3* and *skl1-8* TDNA insertions, shown as inverted triangles, as determined by sequencing from SKL1F and SKL1R primers with the TDNA-specific Lba1 primer. Primers complementary to At*SKL1* exon 4 (SKL1E4) and exon 5 (SKL1E5) are also indicated. C) Analysis of *SKL1* gene structure in homozygous *skl1-8* and *skl1-3* mutants and non-transgenic Arabidopsis (WT) using genomic DNA. Amplification from exon 4 (SKL1E4) to exon 10 (SKL1R) of At*SKL1* was assessed in lanes 1, 3, and 5 (1390 bp). Amplification from exon 1 (SKL1F) to exon 5 (SKL1E5) of At*SKL1* was assessed in lanes 2, 4, and 6 (1195 bp). D) Analysis of *skl1* TDNA alleles from genomic DNA in *skl1-3/skl1-8* variegated Arabidopsis seedling, *skl1-8/skl1-8* albino homozygote Arabidopsis seedling, and *skl1-3*/*skl1-3* variegated homozygote Arabidopsis seedling. The *skl1-3* allele was amplified using SKL1F and Lba1 primers (984 bp–lanes 2, 4, and 6) and the *skl1-8* allele was amplified using SKL1R and Lba1 primer (569 bp–lanes 1, 3, and 5).

**Figure 7:**
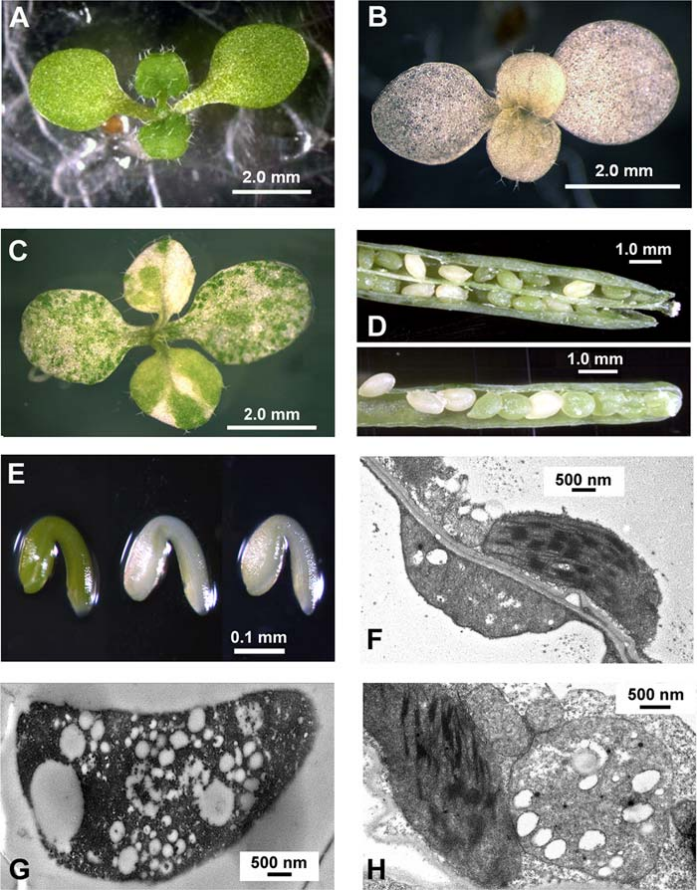
*skl1* TDNA mutant phenotypes. A) 8 day old non-transgenic Arabidopsis seedling, B) 10 day old homozygous *skl1-8* Arabidopsis seedling, C) 10 day old homozygous *skl1-3* Arabidopsis seedling, D) albino seeds from heterozygous *skl1-8* (top) and heterozygous *skl1-3* (bottom) Arabidopsis plants, E) dissected embryos from non-transgenic (left), *skl1-3* mutants (center), and *skl1-8* mutants (right), F) TEM image of interheteroplastidic cells from *skl1-3* green tissue (magnification 15000×), G) TEM image of vesiculated plastid from *skl1-8* albino tissue (magnification 15000×), H) TEM image of intraheteroplastidic cell from *skl1-3* green tissue (magnification 20000×).

The biological role of *SKL1* was probed by mutagenesis. We isolated homozygous T-DNA insertion lines for At*SKL1* (*skl1-3*, *skl1-8*) which further distinguishes the function of this gene from the ancestral SK function. Homozygous T-DNA lines were also isolated for At*SK1* (*sk1-1*) and At*SK2* (*sk2-1*). *Sk1-1* and *sk2-1* were shown by sequencing to harbor TDNA insertions in the first exon of At*SK1* and At*SK2*, respectively. RT-PCR analysis shows that At*SK1* and At*SK2* transcripts do not accumulate in *sk1-1* and *sk2-1* T-DNA mutants, respectively (Figure 6A). Neither of these mutants display any apparent phenotypic defects compared to non-transgenic plants under standard growth conditions. The At*SKL1* T-DNA insertion lines yield severe phenotypes: *skl1-8* is homozygous for an insertion in the 8^th^ exon (Figure 6B) and results in an albino phenotype (Figure 7B), whereas plants homozygous for an insertion in the third intron in *skl1-3* (Figure 6B) have a variegated phenotype (Figure 7C). Plants heterozygous for either the *skl1-8* or *skl1-3* TDNA insertions do not display any apparent phenotypic defects. Unlike *skl1-8*, which is a null mutant, RT-PCR analyses of *skl1-3* seedlings indicate that the AtSKL1 transcript is expressed at low levels (Figure 6A).

Both the variegated and albino phenotypes segregate according to classical Mendelian inheritance for a recessive single locus trait: 193 of 783 (24.6%) progeny counted from self-crossed plants heterozygous for the *skl1-3* insertion displayed the variegated phenotype and 448 of 1742 (25.7%) self-crossed *skl1-8* heterozygote progeny were albino. These segregation ratios are from plants back-crossed to wild-type non-transgenic Arabidopsis. Additionally, pollen from a *skl1-3* heterozygote was crossed into a *skl1-8* heterozygote. Variegated seedlings containing both the *skl1-8* insertion and the *skl1-3* insertion (Figure 6D) were isolated among the progeny of this cross.

During embryogenesis, *skl1-3* and *skl1-8* mutants are completely albino and easily distinguished from non-transgenic and heterozygous individuals (Figure 7D, 7E). Both homozygous *skl1* mutants are soil lethal and will only mature past germination if supplemented with sucrose in the growth media. The cotyledons of *skl1-3* seedlings germinate as albinos and gradually accumulate circular green sectors (Figure 7C). *skl1-3* rosette and cauline leaves emerge with green sectors that appear to overtake albino sectors in a random fashion. There are often marked differences in the patterning and ratio of green:white sectors between leaves of the same *skl1-3* individual.

Transmission electron microscope (TEM) imaging of *skl1-8* mutants shows that chloroplasts with thylakoid membranes do not accumulate in these plants. Instead, *skl1-8* plants accumulate vesiculated plastids lacking internal membrane structure (Figure 7G). Albino tissue sections of *skl1-3* mutants contain *skl1-8*-type vesiculated plastids, whereas *skl1-3* green tissue sections adjacent to albino sections are heteroplastidic, containing a mix of *skl1-8*-type plastids as well as chloroplasts with distinct thylakoid membranes. The mix of plastid types in *skl1-3* green tissue is seen both between (interheteroplastidic) (Figure 7F) and within (intraheteroplastidic) individual cells (Figure 7H).

The degree of albinism and variegation in several previously identified mutants is light-dependent [40]–[41]. Segregating lines of *skl1-3* and *skl1-8* mutants were thus grown under a spectrum of light intensities to assay for photobleaching effects. Growth under a range of 82.5–25 µE had no effect on the degree of *skl1-8* albinism or *skl1-3* variegation. When germinated in the dark, *skl1-3* and *skl1-8* seedlings etiolate to the same degree as non-transgenic seedlings such that they are phenotypically indistinguishable (data not shown). It is interesting to note that *skl1* mutants can accumulate typical stress-related anthocyanins (Figure S4), which are downstream products of the shikimate pathway.

## Discussion

In this study we demonstrate the biological importance of plant *SK* gene duplicate evolution. We have identified two functionally distinct and previously unreported gene families that arose from ancestral plant *SK* gene duplicates. These novel genes, *SKL1* and *SKL2*, lost their ancestral SK activity and acquired novel functions: SKL1 appears to be involved in chloroplast development, and SKL2 acquired a protein–protein interaction domain subject to positive selection among extant plant species. Diversification of gene expression patterns to facilitate developmental requirements and stress responses appears to play an important role in the retention of plant *SK* gene duplicates that maintain their ancestral catalytic activity.

### *SKL1* and *SKL2* Are Ancient *SK* Homologs

Phylogenetic reconstructions of the collection of plant *SK* homologs, from EST assemblies and curated genomic sequences, reveal three distinct clusters of plant SK homologs–*SK*, *SKL1*, and *SKL2*. Although there are significant differences between these families, their global similarities are sufficient to suggest they share a common evolutionary origin. Phylogenetic reconstructions of these families are split into monocot and dicot sub-groups which are derived from moss and pine species. That the respective gene trees match the topology of speciation indicates that these three families are separate orthologous clusters [42]. We have demonstrated biochemically that recombinant AtSKL1 and AtSKL2 do not catalyze SK activity *in vitro*. These recombinant enzymes are stable and purified in the same manner as the active AtSK1 and AtSK2 recombinants. It is also clear from sequence alignments that SKL1 and SKL2 lack several SK substrate binding and catalytic residues.

The taxonomic distribution of genes orthologous to SKL1 and SKL2 was used to estimate the time of origin of these genes. Correlations between the appearance of SKL1 and SKL2 and novel physiological traits in terrestrial plant evolution may provide clues to the specific biochemical functions of SKL1 and SKL2. Although plant SKs are derived from green algae ancestors [26], none of the algal or other microbial sequences retrieved by exhaustive BLAST analyses have the SKL1 PGML or SKL2 CS domains and all contain the prototypical SK consensus sequences, including the RPLL catalytic motif. The presence of conifer and moss orthologs of SKL1 and SKL2, and their absence from all known bacteria and algae, places the emergence of the SKL1 and SKL2 families following duplication of SK genes in a primitive plant species between 400–500 MYA [43]–[44].

It is interesting to note that while the Spruce genome contains distinct orthologs of the plant SK superfamily, the Physcomitrella *SK* homologs differ in their resolution to the SK, SKL1, and SKL2 families. For example, the Physcomitrella SK sequence clusters with microbial SKs and appears as an out-group to plant SKs (Figure 1B). The Physcomitrella genome has only one SK, which contains all of the prototypical SK consensus sequences and lacks the PGML and CS domains. This suggests SK function in Physcomitrella, with respect to regulation and catalytic properties, may be similar to microbial SKs which are also found in single copy. Whereas the Physcomitrella SKL2 is closely related to other plant SKL2 sequences (Figure 1D), the Physcomitrella SKL1 sequence contains partially conserved SK LID and SKL1 PGML consensus sequences (Figure S2). This is reflected in the position of the Physcomitrella SKL1 partway along the branch to the derived plant SKL1 sequences. Despite these differences, it is clear through sequence analysis that early plants such as mosses retained SK activity and had recently acquired *SKL1* and *SKL2* through duplication of an ancestral *SK* gene.

Positive selection pressure resulting in the diversification of amino acid sites involved in substrate binding and catalysis is often the underlying mechanism of enzyme neofunctionalization [45]–[46]. *SKL2* sequences from extant plant species show significant patterns of positive selection. The persistence of positive selection in the *SKL2* family could involve a role in plant-pathogen interactions [47]–[48] or in adaptation to local environments [49]. Positive selection is enriched in the SKL2 CS domain, which likely mediates a protein–protein interaction. The CS domain of plant SGT1 was shown to interact with RAR1 and HSP90 in a complex required for regulation of R protein-mediated disease resistance [50]–[51]. The diversification of SKL2 CS domain sequences among extant plant species could reflect evolving host-pathogen interactions; however this is speculative in the absence of specific knowledge of SKL2 function. Additional genomic SKL2 sequences and biochemical annotation of SKL2 function across extant plant species will be required to specifically address the mechanisms driving the observed signature of positive selection. SKL2 sites aligning to SK P-loop and Walker B motifs are under purifying selection, although the consensus sequences differ substantially from prototypical SKs. It is thus difficult to predict whether SKL2 functions as a kinase.

Following a period of positive selection resulting in the evolution of novel function, purifying selection is expected to act in stabilizing this function [52]. There are strong signatures of purifying selection in the P-Loop, Walker B, and PGML domains of the plant SKL1 family. Diversification of SKL1 sites aligning to the SK shikimate binding and catalytic residues supports the neofunctionalization hypothesis. It is important to note that the modeled structures presented in Figure 4 are template-driven. Loop regions are not accurately predicted using this method, and the models are constrained to match the fold of the template. Consequently, the predicted active site architecture of SKL1 is not useful for docking experiments towards functional annotation. However, that the SK-constrained SKL1 model remains structurally divergent from characterized SK architecture further supports the neofunctionalization model. Although the SKL1 and GntK families are distantly related, they are similar in their enriched conservation of plant and microbial SK P-Loop and Walker B ATP binding sites. Conservation of these sites under purifying selection suggests SKL1 enzymes catalyze a phosphorylation reaction but have acquired specificity for a novel substrate. SKL1 has conserved some shikimate binding sites which suggests the substrate of SKL1 is structurally similar to shikimate. Although SKL1's cognate substrates are unknown, the solvent exposed PGML motif could be involved in allosteric regulation.

*SKL1* and *SKL2* show distinct signatures of selection and structure, but are similar in having lost their ancestral SK activity and acquiring functions that resulted in their retention for ∼500 million years of evolution. In this sense it appears that *SKL1* and *SKL2* are neofunctionalized *SK* duplicates. However, determining the specific biochemical functions of SKL1 and SKL2 will allow a more comprehensive understanding of the evolutionary mechanisms driving their diversification. SKs have also been implicated in enzyme neofunctionalization in bacteria. Unlike most bacteria, *E. coli* has two SK isoforms–SKI (AroK) and SKII (AroL). These isoforms are 33% identical and are structurally similar. However, *E. coli* AroK has approximately 100-fold lower affinity for shikimate (K_m shikimate_ = 20 mM) than *E. coli* AroL (K_m shikimate_ = 200 uM) [53]. This difference in shikimate binding affinity is unique to *E. coli*, as both the AroK-type MtSK and the AroL-type EcSK share shikimate K_m_ values comparable to *E. coli* AroL. Also unique to *E. coli* is the role of AroK in conferring resistance to mecillinam [54]. These authors suggest that *E. coli* AroK has acquired a second activity related to the regulation of cell division. Taken together, these findings demonstrate the utility of the P-loop module and the NMPK fold as a scaffold for functional innovation.

### *SKL1* and Chloroplast Biogenesis

The classic segregation ratios and the similarity of the *skl1* phenotypes suggest they are specific to the AtSKL1 locus, and not a result of additional genomic TDNA insertions. The accumulation of anthocyanins in *skl1-8* mutants indicates that the albino phenotype is not due to a general restriction of the shikimate pathway and further supports the neofunctionalization model. The striking *skl1* phenotypes and the predicted localization of SKL1 to the chloroplast stroma collectively indicate the *in vivo* function of SKL1 is required for chloroplast biogenesis.

The variegated *skl1-3* phenotype is unique among the known plant variegation mutants [55]–[57] in that the seedlings germinate as albinos with green sectors expanding with time independently of light intensity. This phenotype could be explained in part by the attenuated expression of SKL1 in this mutant line, as shown by RT-PCR analysis. The *skl1-3* TDNA insertion fragment could be transcribed and subsequently removed by an inefficient splicing event, resulting in decreased SKL1 transcript levels. The heteroplastidic phenotype of the *skl1-3* mutants suggests genetic heterogeneity between chloroplasts [58] sets plastid-specific abundance threshold levels of SKL1 required for the progression of chloroplast biogenesis. This is similar to the variegation model proposed for *var1/2* mutants. VAR1 and VAR2 encode Ftsh proteins [59]–[62], which are ATP dependent metalloproteases involved in turnover of the D1 reaction centre protein of photosystem II following photo-oxidative damage [63]. The mechanism of variegation in *var1/2* is thought to involve an abundance threshold of oligomeric AtFtsh isoform complex formation required for chloroplast development [64]. Many of the reported albino plant mutations arise from disruptions of the non-mevalonate plastid isoprenoid biosynthetic pathway [65]–[70] and plastid protein and small molecule import/transport [71]–[73]. Albino plants have also been isolated from the well-characterized phylloquinone [74] and chlorophyll [75]–[77] biosynthetic pathways. It is unlikely that SKL1 functions in carotenoid biosynthesis, as the *skl1* mutants are not due to photobleaching effects.

A common characteristic of plant albino and variegated mutants is the disruption of biosynthesis or transport of small molecules or peptides required for chloroplast functions. The diversification of the SKL1 active site could facilitate phosphorylation of a protein. The variegated *skl1-3* phenotype and the vacuolated plastids of *skl1-8* resemble the *ppi2* and *apg2* mutants, both of which are involved in protein import into the chloroplast. SKL1 could thus function as a regulator of plastid protein import complexes. It is also possible that SKL1 performs a crucial structural function or protein phosphorylation reaction required for assembly or stability of the photosynthetic apparatus. The lack of purifying selection in the SKL1 region aligning to the SK LID domain suggests SKL1 could adopt an open disordered conformation sufficient to accommodate a protein substrate. However, the degree of sequence similarity between SKL1 and other SK sequences suggests that SKL1 is more likely a small molecule kinase.

Retrograde signaling, wherein plastid- or mitochondria-originated signals direct nuclear transcription, has been shown to be necessary for chloroplast development [78]. It is possible that SKL1 is a regulator of these processes or participates directly in a biosynthetic pathway linked to retrograde signaling. In this model, loss-of-function mutations of SKL1, such as *skl1-8*, would result in the loss of a signal necessary for the continuation of the nuclear-encoded chloroplast development program. A conceptually similar scenario was reported for mammalian glucokinases, where the activity of the mitochondrial-localized enzyme is linked to a signaling cascade that regulates programmed cell death in response to glucose levels [79].

### Regulatory Neofunctionalization of *SK* Duplicates and Metabolic Regulation

The phylogenetic distribution of the plant SK family shows that duplicates of these genes are often retained, with the number of paralogs varying between species. Given the limited availability of sequence data it is difficult to assess the generality of this observation. For example, there is no additional moss *SK* at the base of the plant *SK* clade (Figure 1). In vascular plants, it appears that regulatory neofunctionalization of *SK* paralogs imparts a selective advantage by refining gene expression in a lineage-specific manner. For example, differential expression of the three rice *SK* inparalogs is involved in panicle organogenesis and floral organ development [6]. The Arabidopsis *SK* inparalogs, which have similar kinetic profiles, have also acquired distinct expression patterns. At*SK2* is typically expressed at a steady state in green tissues throughout development and is only mildly induced under specific challenges, such as *P. infestans* infection. Conversely, At*SK1* is typically expressed near or below background levels throughout development and is only highly induced under heat stress and specifically in petals and stamens, late embryogenesis, and advanced stages of pollen production and receptive stigmas. We refer to this gene expression divergence as regulatory neofunctionalization, as opposed to subfunctionalization, as ancestral SKs such as those from microbes clearly do not possess regulatory elements directing expression to floral organs.

The selective advantage of regulatory sub- or neofunctionalization among metabolic isozymes remains unclear. Small changes in protein structure could function to optimize activity in distinct environmental or cellular conditions such as pH, or ion and substrate abundance. An alternative model suggests that concerted divergence of gene expression can result in neo- or subfunctionalized inparalogs forming separate co-expression networks [80]. In this view, we can derive a model with AtSK2 as the main contributor to carbon flux through the shikimate pathway in vegetative tissues and AtSK1 functioning primarily to increase carbon flux to specific metabolite pools in response to environmental stress or tissue-specific developmental requirements. For example, the tomato *SK* transcript is induced approximately 17-fold by fungal elicitors [4], which could result in re-direction of carbon flux to phytoalexin biosynthesis. Interestingly, unlike other fungal-elicited tomato shikimate pathway transcripts, the tomato SK induction pattern is not delayed, which supports a role in direct response to the pathogen challenge [4]. Mechanisms for plant SK paralog-specific metabolic flux re-distribution could involve hitherto undescribed branch points of the shikimate pathway, additional functions for plant SKs, or differences in metabolic machinery closely associated with plant SK inparalogs [81]–[82]. The diversity of SK paralog copy number between plant species suggests extrapolation of regulatory network topologies from model organisms to unsequenced crop species may be substantially more difficult than anticipated. Deciphering the contribution of *SK* gene expression dynamics to changes in metabolic flux will require further study.

Studies of gene duplicate fate are complicated in most enzyme families by extreme amplification. For example, it has been predicted that over 10,000 CYP450's comprising ∼100 families have arisen by gene duplication among extant higher plant species [83]. The plant *SK* superfamily described here allows the study of gene duplicate fate in a small and ancient group of duplicates. We have identified novel orthologous clusters of ancient and physiologically important plant genes and report novel chloroplast biogenesis mutants. The high degree of sequence similarity between SKL1 and ancestral SKs underscores the importance of validating bioinformatics-based functional annotation. High-throughput biochemical techniques for reliably matching enzymes of unknown function with their cognate substrates will be important for comprehensive mapping of metabolic pathways in plants.

Whereas ancient *SK* gene duplicate retention appears to involve the acquisition of novel gene product function, it appears that differential regulation of plant *SK* paralogs during developmental processes and stress responses is important for the retention of more recent *SK* duplicates. An understanding of how plant SKs contribute to metabolic regulation in response to dynamic cellular and environmental conditions will be relevant to the rational engineering of plant metabolism and deciphering the relationship between shikimate pathway output and plant development and stress response. These findings demonstrate that plant SKs have played an important role as scaffolds for functional innovation as well as the diversification of metabolic regulation.

## Methods

### SK Homolog Identification and Phylogenetic Reconstructions

BLASTp using functionally the characterized tomato and rice SKs against the NCBI non-redundant (NR) database [84] with a significance threshold of E = 1e-20 returned sequences from beech (*Fagus sylvatica*), grape (*Vitis vinifera*), cucumber (*Cucumis sativus*), moss (*Physcomitrella patens*), and *Arabidopsis thaliana*. BLASTp against the Arabidopsis genome through TAIR [85] identified an additional protein annotated as ‘shikimate kinase-related’. These SK homologs were used as tBLASTn queries (cut-off P = 10e-30) against the TIGR gene indices [86], composed of expressed sequence tags (ESTs) assembled into contiguous protein coding sequences. This analysis returned an additional 37 plant SK homologs, including sequences from maize (*Zea mays*), barley (*Hordeum vulgare*), sorghum (*Sorghum bicolor*), sugercane (*Saccharum officinarum*), wheat (*Triticum aestivum*), spruce (*Picea glauca*), poplar (*Populus trichocarpa*), soybean (*Glycine max*), cotton (*Gossypium hersitum*), potato (*Solanum tuberosum*), and medicago (*Medicago trunculata*). BLASTp analysis against the recently released *P. patens* genome [87] identified 4 SK homologs with E<10e-30 for at least one of the query sequences retrieved from NCBI-NR or the TIGR gene indices. Sequences retrieved from BLASTn analysis with the TIGR TC collection were manually evaluated to filter sequences with low depths of coverage (less than two independent ESTs per base) and fragments unsuitably short for multiple sequence alignment.

Multiple sequence alignments were generated with MAFFT [88] using the slow, iterative method (FFT-NS-i) and manually edited by eye in MEGA 4.0 [89]. SK, SKL1, SKL2, and GntK families were individually aligned first to guide the composite alignment. Distances, defined as the number of amino acid substitutions per site, were calculated in MEGA 4.0 using the Neighbor Joining algorithm with the Poisson Correction distance model [90] and pairwise deletions for the unrooted tree containing the SK, SKL1, SKL2, and GntK families. Bootstrap consensus scores were generated from 1000 iterations. Distances between clades were calculated in MEGA 4.0 using between group averages with the amino acid Poisson correction model, uniform rates among sites and lineages, pairwise deletion, and 500 bootstrap iterations to calculate standard error.

### PAML Selection Analysis

Nucleotide sequences for the plant SK, SKL1, and SKL2 families were aligned by codons with MAFFT [88] using the slow, iterative method (FFT-NS-i) and manually edited by eye in MEGA 4.0 [89]. PAML version 4.0 [36] analyses were performed using the codeml module. Models M0 (one-ratio), M1 (neutral), M2 (selection), M3 (discrete), M7 (beta), and M8 (beta+ω) were analyzed. MEGA 4.0 was used to generate a neighbor joining tree using the composite maximum-likelihood distance correction for the tree file. The following parameters settings were used in the control file: CodonFreq = 2, clock = 0, aaDist = 0, model = 0, cleandata = 0, fix_kappa = 0, fix_omega = 0. *P. patens* sequences, and sequences lacking any of the functional domains described in this paper, were not included in the PAML analysis. Estimates of substitution saturation were calculated as per [39].

### Structure Models

AtSK2 and AtSKL1 model coordinates were generated using Phyre [28] with MtSK complexed with ADP (PDB:1L4U) as a template. The AtSK2 and AtSKL1 coordinates were aligned to MtSK complexed with ADP and shikimate (PDB:2IYQ) using the DaliLite server [91]. Structure figures were generated using PyMOL [92].

### Construct Design and Protein Purification

Appropriate construct lengths for AtSK homologs were determined from secondary structure prediction, with the majority of the putative cTP regions truncated and all downstream regions with predicted secondary structure retained. The clones were amplified from Arabidopsis cDNA, prepared as per RT-PCR analysis from non-transgenic seedlings. These PCR products were ligated into pET15b using T4 DNA ligase and transformed into BL21 *E. coli* cells. Recombinant proteins were purified as previously reported [93]. Primers used to clone the recombinant constructs are listed in Table S4.

### Enzyme Kinetics

Enzymatic activity of recombinant proteins was assayed by monitoring the oxidation of NADH (ε = 6200 M^−1^ cm^−1^) at 340 nm coupled to pyruvate kinase (EC 2.7.1.40) and lactate dehydrogenase (EC 1.1.1.27) following the release of ADP from ATP by the shikimate kinase-catalyzed reaction [22]. The assay was carried out at 25°C in a 1 mL mixture containing 100 mM Tris-HCl, 1 mM phosphoenolpyruvate, 0.1 mM NADH, 2.5 mM ATP, 2.5 mM shikimate, 3.6 units of pyruvate kinase, and 5.5 units of lactate dehydrogenase. Saturation kinetics studies were carried out at pH 8.0, determined to be the optimal pH for shikimate kinase activity. The apparent Km values for shikimate were determined using a fixed [ATP] of 2.5 mM and a range of [shikimate] from 12.5 uM to 1.0 mM. Apparent ATP Km values were determined using a fixed [shikimate] of 2.5 mM and a range of [ATP] from 12.5 uM to 1.6 mM. Saturation curves were determined using triplicate values from the linear portion of the reaction curves. Kinetic parameters were calculated as previously described [94].

### Plant Growth and Genotyping

Arabidopsis TDNA lines used in this study were obtained from the Arabidopsis Biological Resources Center. Seedlings were grown on Petri plates under 24 h light at 22°C on 1× MS salts (Gibco BRL), pH 5.7, with 1.5% (w/v) sucrose and 1% (w/v) phytagar. Arabidopsis genomic DNA was extracted by homogenizing seedlings with mortar and pestle in liquid nitrogen. Extraction buffer (200 mM Tris-HCl pH 8.0, 250 mM NaCl, 25 mM EDTA pH 8.0, 0.5% SDS) was added to each sample. One half volume of phenol∶chloroform∶isoamyl alcohol (24∶24∶1) was added, vortexed and centrifuged for 10 minutes at 14000 g. Supernatant was collected and DNA was precipitated with an equal volume of isopropanol. DNA was pelleted by centrifugation at 14000 g for 10 minutes, allowed to air dry and resuspended in sterile water. For each putative TDNA insert-containing line, at least two PCR reactions were conducted, where separate primer pairs were used for genotyping. Specifically, a forward and reverse gene-specific primer pair, and a T-DNA left-border-specific primer and one gene specific primer combination, were used to screen each line. Genotyping primer sequences are listed in Table S4. All genotyping PCR reactions were performed using Tsg polymerase (BioBasics) under standard conditions. Lba1/gene-specific PCR products were gel purified (Qiagen PCR Purification Kit) and sequenced to verify the location of the T-DNA insert. Light intensity gradients were established using layers of cheesecloth covering the Petri plates containing Arabidopsis seedlings and measured using a LI-COR photometer (model LI-185B).

### RT-PCR

RNA was extracted from 10-d-old seedlings grown under 24 h light using TRIzol Reagent (Invitrogen) according to the manufacturer's instructions. cDNA was prepared using 200 units of SuperScript II (Invitrogen) reverse transcriptase, 1 ug total RNA, and 0.5 µg Oligo(dT)_20_ (Invitrogen). Primers used for RT-PCR are listed in Table S4. Tsg polymerase (BioBasics Inc) was used for all RT-PCR reactions under standard conditions. RNA extractions and RT-PCR amplifications were performed in triplicate yielding the same results.

### TEM Images

Leaf tissue from Arabidopsis seedlings at the Boyesian growth stage 1.2 [95] were first cut into ∼1 mm^2^ pieces and fixed in 2.5% (v/v) glutaraldehyde in 0.1 M sodium cacodylate buffer (pH 7.2) overnight under vacuum at 25 psi. The tissue was then washed twice for 1 h in 0.1 M sodium cacodylate (pH 7.2) and post-fixed with 2.0% (v/v) osmium tetroxide in cacodylate buffer for 3 h at 4°C. Following two more washes for 1 h in cacodylate buffer the tissue was dehydrated through a graded alcohol series for 1 h with 5%, 10%, 20%, 30%, 50%, 60%, 80%, 90%, and 100% anhydrous ethanol and subsequently left overnight at 4°C in 100% anhydrous ethanol. The tissue was embedded in LR White acrylic resin (Polysciences Inc.), which was allowed to polymerize for 24 hours at 65°C. Polymerized blocks were subsequently sectioned using a Reichert-Jung ultramicrotome, mounted on Formvar-coated 200-grid supports and stained with lead citrate and uranyl acetate before viewing on a Philips 300 transmission electron microscope.

## Supporting Information

Figure S1 A) Full-length alignment of *E. coli* GntK [16]–[17] with AtGntK and MtSK. Key binding residues are marked with *, B) tertiary alignment of *E. coli* GntK [1KNQ] shown in red with MtSK [1L4U] shown in cyan. Ribbon diagram generated using PyMOL [91].
(0.27 MB DOC)

Figure S2 Amino acid multiple sequence alignments for A) Arabidopsis SK homologs aligned to bacterial SKs, B) plant SK family alignment, C) plant SKL1 famiy alignment with the PGML domain indicated, D) plant SKL2 family alignment with the CS domain indicated, E) the Arabidopsis SK homologs with the AtSKL2 CS domain and the AtSKL1 PGML domain highlighted in red. N-terminal cTP regions have been excluded from the alignments. Sites marked with * in A) indicate a direct role in SK substrate binding or catalysis as determined from microbial SK crystal structures.
(1.51 MB DOC)

Figure S3 Multiple sequence alignment of SKL2 CS domains. Expect values for Pfam CS domain model PF04968 are indicated.
(0.06 MB DOC)

Figure S4 Anthocyanin accumulation in 8 days old Arabidopsis *skl1-8* mutant seedling. Scale bar = 1.0 mm.
(0.44 MB DOC)

Table S1 Accession numbers for plant SK homolog protein sequences retrieved from NCBI-nr and the Physcomitrella patens genome resource (www.cosmoss.org).
(0.05 MB DOC)

Table S2 Predicted cTP sequences for SK, SKL1, and SKL2 plant sequences. The optimal cut-off for ChloroP cTP detection significance scores is 0.5 [28].
(0.06 MB DOC)

Table S3 Model M2a posterior probability scores for positively selected sites in the SKL2 family.
(0.07 MB DOC)

Table S4 Primer sequences.
(0.03 MB DOC)
