# Supplementary figures and images for: Evolutionary Diversification of Plant Shikimate Kinase Gene Duplicates
Source: PLoS Genet. 2008 Dec 5;4(12):e1000292. doi: 10.1371/journal.pgen.1000292 (PMC2593004; doi:10.1371/journal.pgen.1000292)

**A**

**
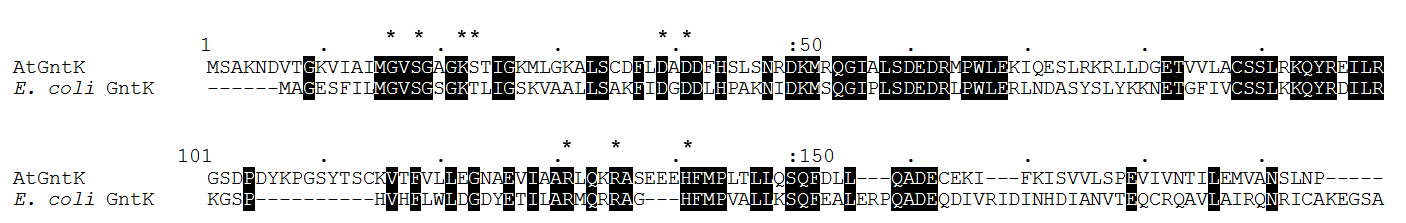
**

**B**

**
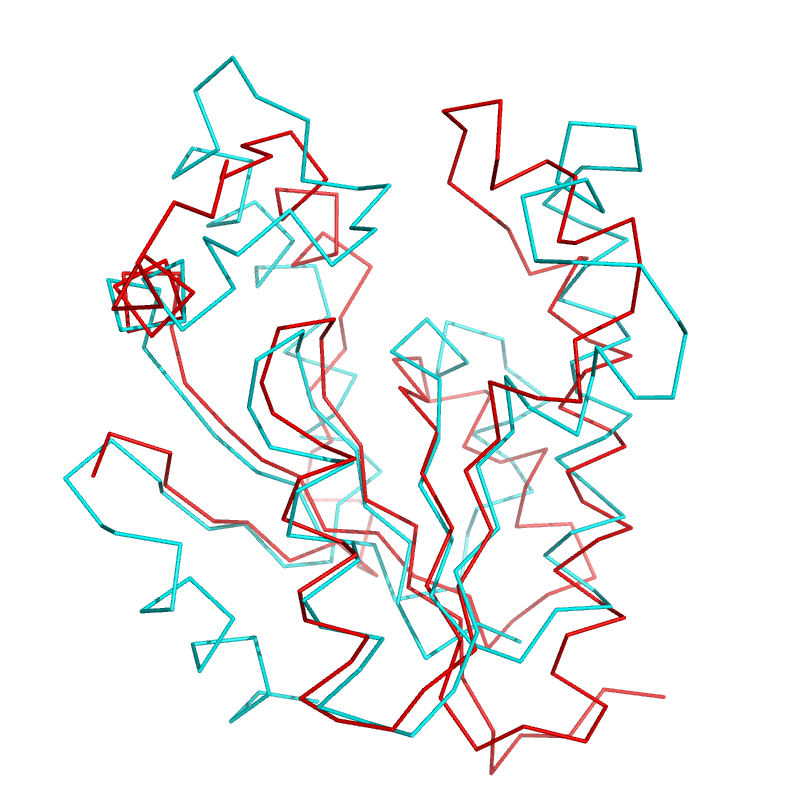
**

Supplement: Figure S1 — A) Full-length alignment of E. coli GntK [16]–[17] with AtGntK and MtSK. Key binding residues are marked with *, B) tertiary alignment of E. coli GntK [1KNQ] shown in red with MtSK [1L4U] shown in cyan. Ribbon diagram generated using PyMOL [91]. (0.27 MB DOC) [file pgen.1000292.s001.doc]

**A**

**
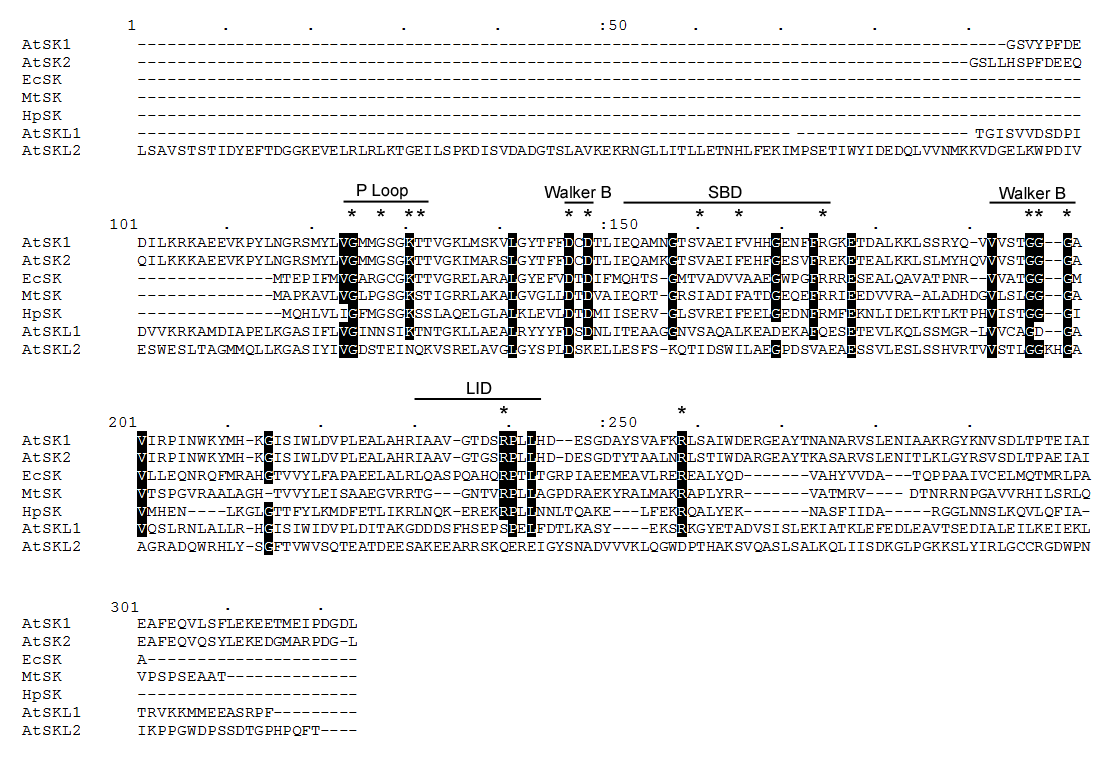
**

**B**


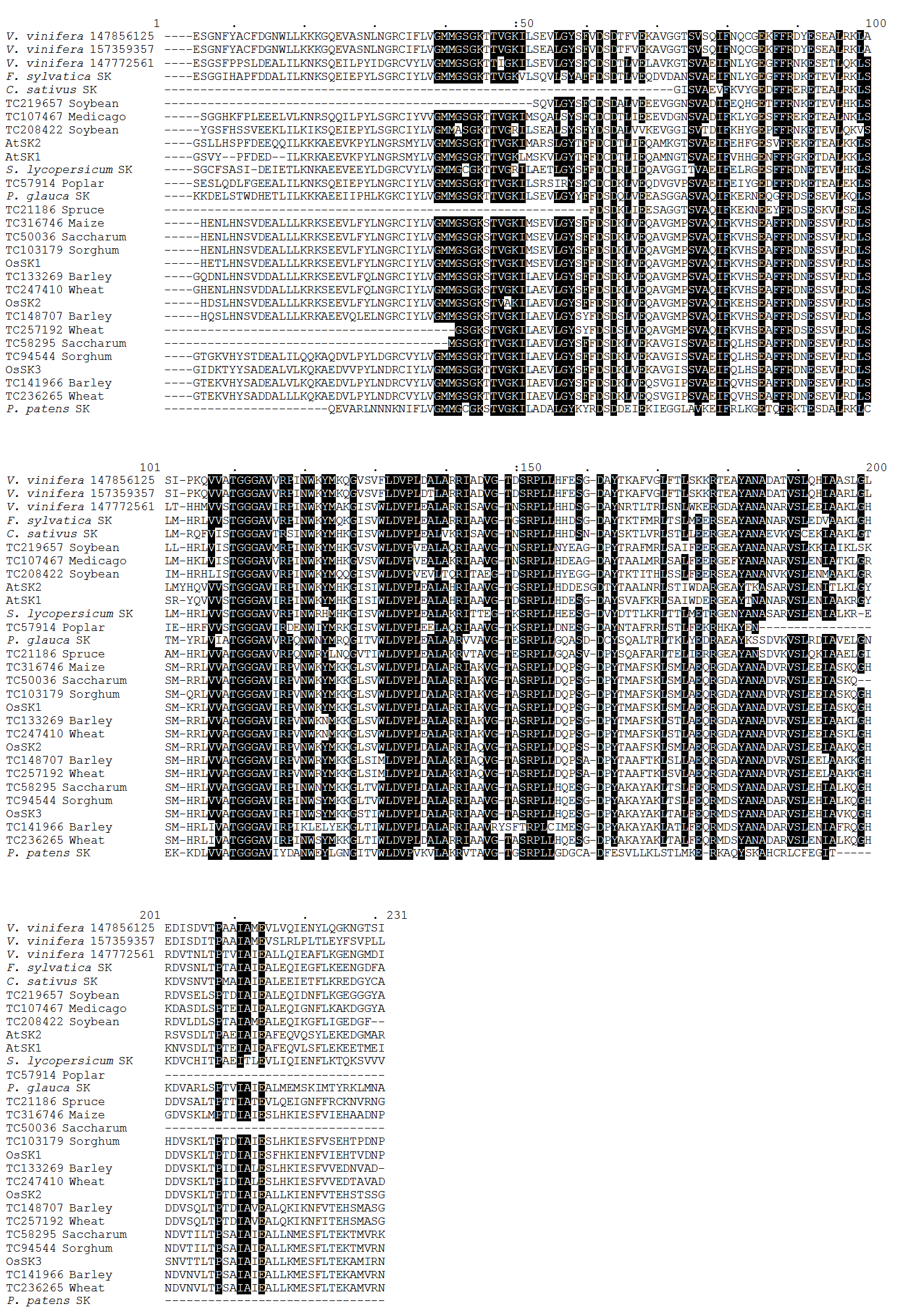


**C**

**
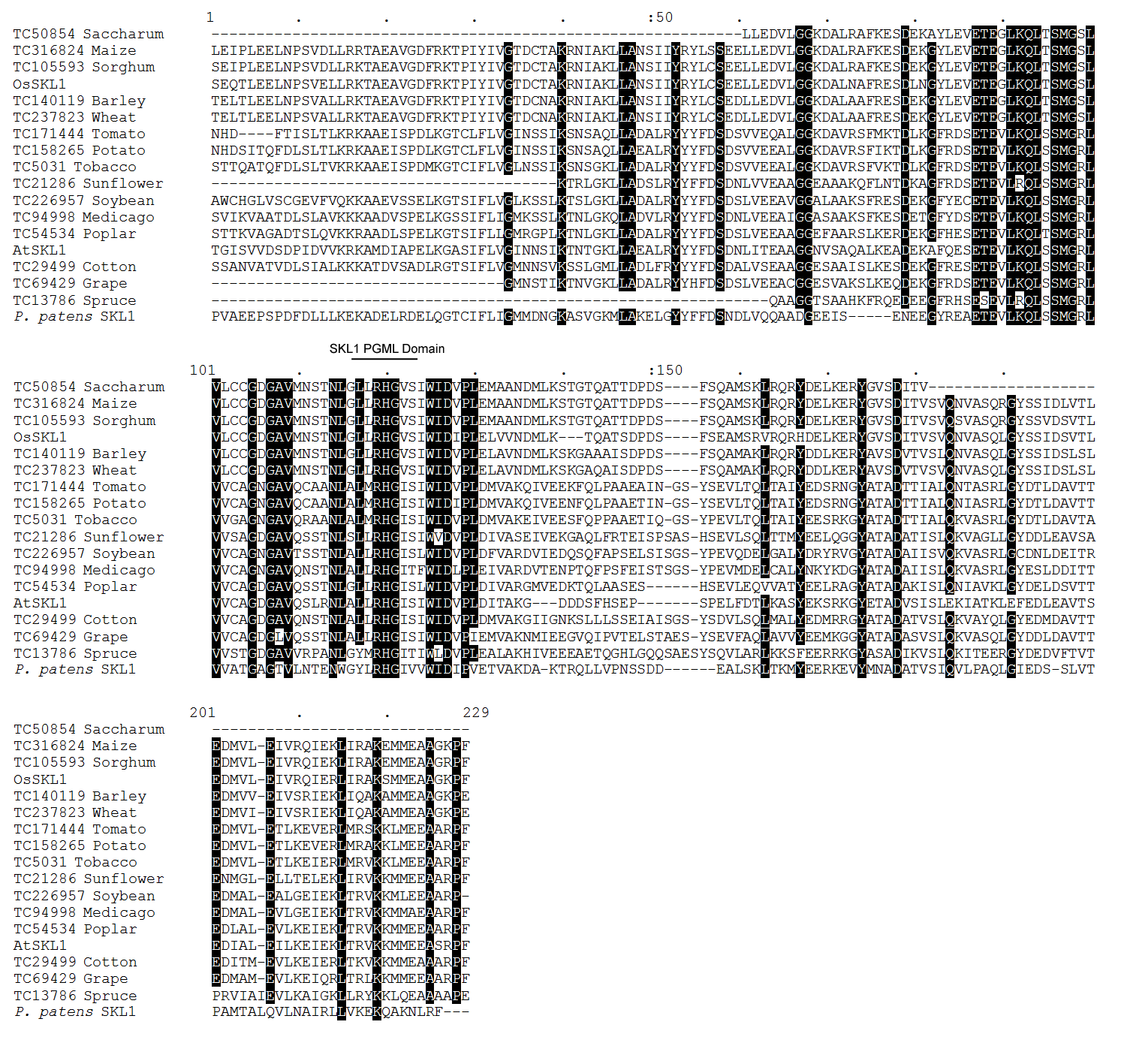
**

**D**

**
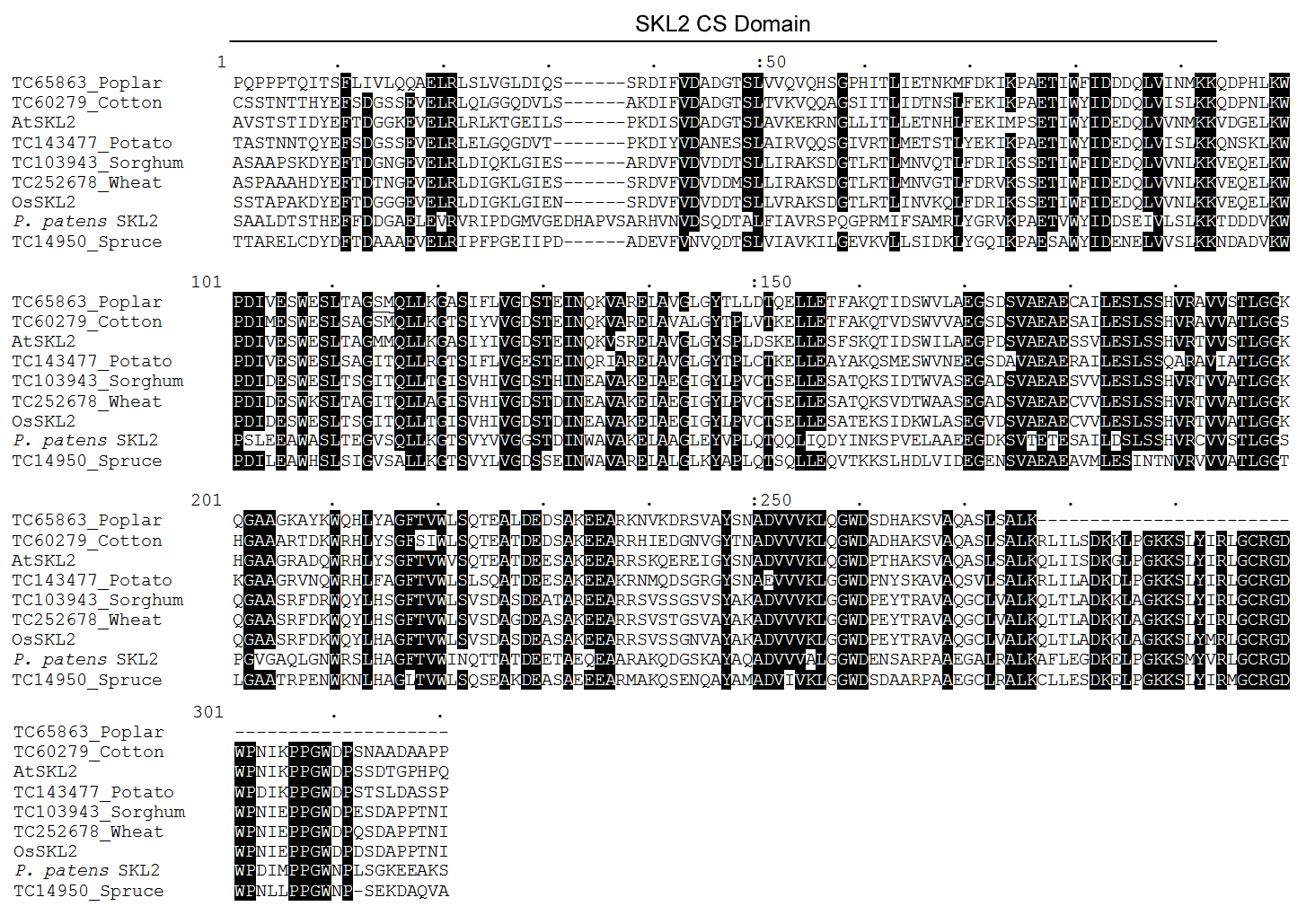
**

**E**


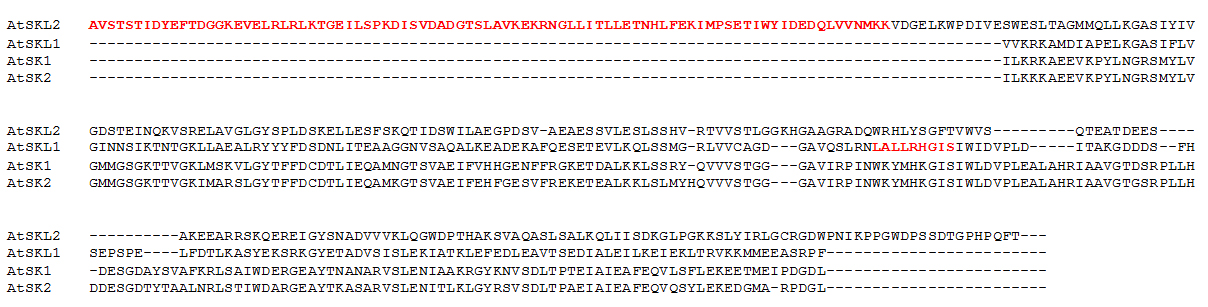

Supplement: Figure S2 — Amino acid multiple sequence alignments for A) Arabidopsis SK homologs aligned to bacterial SKs, B) plant SK family alignment, C) plant SKL1 famiy alignment with the PGML domain indicated, D) plant SKL2 family alignment with the CS domain indicated, E) the Arabidopsis SK homologs with the AtSKL2 CS domain and the AtSKL1 PGML domain highlighted in red. N-terminal cTP regions have been excluded from the alignments. Sites marked with * in A) indicate a direct role in SK substrate binding or catalysis as determined from microbial SK crystal structures. (1.51 MB DOC) [file pgen.1000292.s002.doc]

**
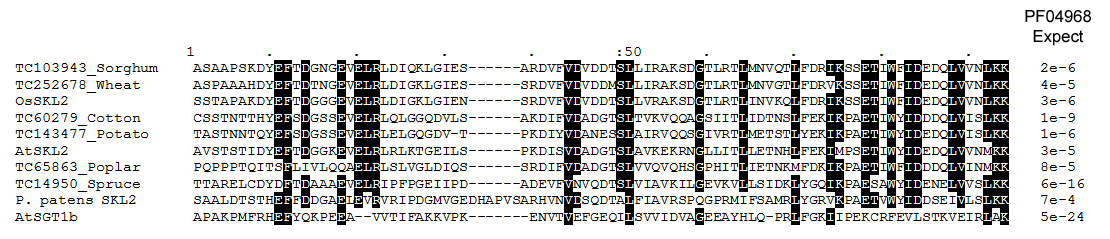
**

Supplement: Figure S3 — Multiple sequence alignment of SKL2 CS domains. Expect values for Pfam CS domain model PF04968 are indicated. (0.06 MB DOC) [file pgen.1000292.s003.doc]

**
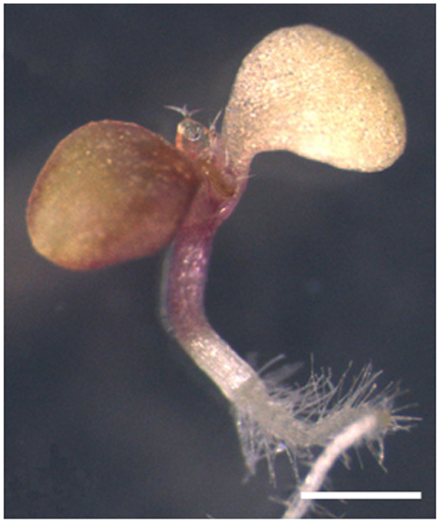
**

Supplement: Figure S4 — Anthocyanin accumulation in 8 days old Arabidopsis skl1-8 mutant seedling. Scale bar = 1.0 mm. (0.44 MB DOC) [file pgen.1000292.s004.doc]
