# Supplementary material for: Evolutionary Diversification of Plant Shikimate Kinase Gene Duplicates
Source: PLoS Genet. 2008 Dec 5;4(12):e1000292. doi: 10.1371/journal.pgen.1000292 (PMC2593004; doi:10.1371/journal.pgen.1000292)
Supplement: Table S1 — Accession numbers for plant SK homolog protein sequences retrieved from NCBI-nr and the Physcomitrella patens genome resource (www.cosmoss.org). (0.05 MB DOC) [file pgen.1000292.s005.doc]

| **Species/Sequence Name** | **Accession** |
| --- | --- |
| *Arabidopsis thaliana* SK2 | NM_202986 |
| *Arabidopsis thaliana* SK1 | NM_201778 |
| *Oryza sativa* SK1 | AB188834 |
| *Oryza sativa* SK2 | AB188835 |
| *Oryza sativa* SK3 | AL606455 |
| *Vitis vinifera* SK1 | AM441847 |
| *Vitis vinifera* SK2 | AM431791 |
| *Vitis vinifera* SK3 | CU459368 |
| *Lycopersicon esculentum* SK | BT012939 |
| *Cucumis sativus* SK | AY596190 |
| *Fagus sylvatica* SK | DQ166524 |
| *Nostoc* sp. PCC 7120 SK | BA000019 |
| *Anabaena variabilis* ATCC 29413 SK | CP000117 |
| *Chlamydomonas reinhardtii* SK | XM_001690350 |
| *Helicobacter pylori* SK | AE000511 |
| *Erwinia crysanthemi* SK | X14777 |
| *Escherichia coli* AroL | X04064 |
| *Escherichia coli* AroK | ECOAROK |
| *Mycobacterium tuberculosis* SK | CP000717 |
| *Physcomitrella patens* SK | Phypa_5592 |
| *Picea sitchensis* SK | EF085042 |
| *Arabidopsis thaliana* SKL1 | NM_113602 |
| *Oryza sativa* SKL1 | NM_001048285 |
| *Physcomitrella patens* SKL1 | Phypa_166136 |
| *Arabidopsis thaliana* GntK | NM_127231 |
| *Oryza sativa* GntK | AK240881 |
| *Physcomitrella patens* GntK | Phypa_128786 |
| *Vitis vinifera* GntK | CU459227 |
| *Escherichia coli* GntK | AP009048 |
| *Physcomitrella patens* SKL2 | PP020019201R |
| *Oryza sativa* SKL2 | NM_001072025 |
| *Arabidopsis thaliana* SKL2 | NM_129102 |
| *Physcomitrella patens* Unknown | Phypa_75102 |
