# Supplementary material for: Evolutionary Diversification of Plant Shikimate Kinase Gene Duplicates
Source: PLoS Genet. 2008 Dec 5;4(12):e1000292. doi: 10.1371/journal.pgen.1000292 (PMC2593004; doi:10.1371/journal.pgen.1000292)
Supplement: Table S2 — Predicted cTP sequences for SK, SKL1, and SKL2 plant sequences. The optimal cut-off for ChloroP cTP detection significance scores is 0.5 [28]. (0.06 MB DOC) [file pgen.1000292.s006.doc]

| **Protein Name** | **Score** | **Predicted cTP Sequence** |
| --- | --- | --- |
| PhyscoSKL2 | 0.573 | MASLSVRGATLYSATSLWAPLVSPRCASPNRSSVRFADRHSLLCTETKANWKSSNSVSKYLRRSVAKSFV |
| AtSKL2 | 0.594 | MAAFASGLAIIFNSPSLNPVTTQATFLSSNRIRSSPRVFSGFHSLRRRGFRRFSQNVIPDRFNSFSCNC |
| TC14950_Spruce | 0.532 | ASFPAITQSRDTRSILVAFKCTHNYQFQHSLLSNSHSLSFLFAPTSLHRLKVSNKHGIGLFNICSARL |
| TC65863_Poplar | 0.521 | QKNGHHHHHYSDSFLPLPKPHQNSTIILSLKYPLSPFPKPISLPSPICLFQFLPLNQTMRTALSTDTAAS |
| TC60279_Cotton | 0.574 | ASAAASASSMLCLSLQNPTKTLHFSIKTHSFHFLKPQLPAFRRYSTGVTPISPLRGISCNCSSTNTTHYEFSDGSS |
| TC143477_Potato | 0.557 | ASSSSVALSFLYQNPIKASLSIPKAFSPYSKLTFPSISSFYSAIPRLDYSNFKKINPCLHCSNLPTAS |
| TC103943_Sorghum | 0.578 | RIGAGAAAAANMAACSSVPCSFPTKPDPHRRGRSSHLPRLPAATQFRRFRSLSAAAAASSSASRLRPRASVSAS |
| OsSKL2 | 0.576 | MLASTCFSAPPPSSSSPSIPTHLATLCCCFRPPARPPWPRSLLLLGAFPPPTRPLPRASVSSS |
| TC252679_Wheat | 0.575 | MAAMSIAATATCCSSSFPANPSQHIATYSRAPGLPLPRPAWRRSLLAASPPASRLRLLPRTSLSAS |
| AtSK2 | 0.453 | MEAATVQRFQYSSWNDLRNFEGKPRGSLRYNTQRIKEDKRFRVVALTLDKRRDHRLRSVSDKNSSALLET |
| AtSK1 | 0.471 | MEAAITQRIQYPSWVDCRKVECKPQRGSLRYSQQVKVDRRFRGLSLARLQPERRNDQRRAVSPAVSCSDNNSS |
| TC30351_Spruce | 0.467 | AVFSMELGRIELGSSWKVQSHTYCPLQDARSRKKMLSVKFAGQWKGGELQRCNNLQVVIPEKRSVLNDARLPS |
| Tomato_SK | 0.483 | MEARVSQSLQLSSWINSDKVVRKPSGLLRFSEKWNEKPRHRVVVSCHLQPRKAAHSDRRVQLKVSCS |
| OsSK1 | 0.46 | MEAGVGLALQSRAAGFGGSDRRRSALYGGEGRARIGSLRVAEPAVAKAAVWARGSKPVAPLRAKKSS |
| OsSK2 | 0.522 | MEARAGLAMQSRAAVGVGAGPGVGRRGRAVIRVGKRPTAASLRVGGPAGPAAAKPLAPLYCLKASRGHDS |
| OsSK3 | 0.534 | MDAGVGLRAKPGAWAGLGNPRRSSTARVPVRFAVEKFAQPLVLGSDRRSCGAKLKVSCSR |
| TC316746_Maize | 0.517 | EAGGVGLALQTRAAAFGSGQRRGGLQSPIGRLRVAEPAGAAVAVRVRGSKPVVPLRAKKS |
| TC103179_Sorghum | 0.549 | HISATATGYHLPPAPSASLDLRIAPRLSRSRLVAASPLPAQQQQQQRMEAGLALQTRAAGFGS |
| TC50036_Saccharum | 0.502 | AAEHHPASGERAMEAGGVGLALQTRAAGFGSGRRRGGLQSPSGSLRVADPSGAAVAVRARGS |
| TC133269_Barley | 0.512 | EAGAGLALQSRAAGFGSGRRRSAMYGGESGARMVSLRVGDQVGSPAAVRARGAKPVVPLRAKKSS |
| TC247410_Wheat | 0.528 | EAGVGLALQSRAAGFGSGRRRRATYGGESRARTVSLRVSDLVGSPAAVRARGAKPVVPLRAKKSS |
| TC148707_Barley | 0.534 | EAAAGVAMQSRAVGVAGTGSCGRRSGDGRARPGSLRVGGPAAAPVLRARGARPADPLCCLKTSRGHQS |
| TC141966_Barley | 0.45 | DAGVCLRPRPRAWAGRREQQEFPPAILPAARLAVAADQNPARRPLVLRSGAGRRSADPIRGAARLKGLCCQKS |
| TC236265_Wheat | 0.496 | DAGVGLRPRPRAAWAGRRKPQGFPPATVPAARLDQNPARRPLVLRPDAIRGASLKGLRCHKSAGT |
| TC94544_Sorghum | 0.461 | EASVGVRAPARGRAWAGIEKPHGAYSVRVPPVRLTADRLRLPPPRRLVLGADPWRTAGPALRPAKLRVSCS |
| TC57914_Poplar | 0.537 | WIDSYKFPRKPTSSLRFSGRFKEQKRLQVFVSAQFRPVRDENRHRQASFEVSCSCNNSQV |
| TC208422_Soybean | 0.554 | EAQAVQVLRYSATLHSNNPKLEKTGTNGSLRMFGGFKKQLFVSSKLQSAKPSANIRRRTASLVVACS |
| TC107467_Medicago | 0.439 | KVSLEVACSYNNIPASTLESGGHKFPLEEELVLKNRSQQILPYLSGRCIYVVGMMGSGKTTVGKIMSQALSYS |
| AtSKL | 0.56 | MEIFSASASLTLTGFVPRLLPLLSPQARTTLCKPLLSSSSTRLISCHSRIAPSRSLADQS |
| OsSKL | 0.577 | MAMAMAAMRGAAASLGPGAVSWNSKRFFLSSSSTTSRPHRRRLRAFPSSEQTLEELNPSVELLRK |
| TC171444_Tomato | 0.474 | KVVQLAQPFVVPTFCHHLKPPRAVSKHNHD |
| TC158265_Potato | 0.48 | VVQLAQQLWPLVGHSRPTTICPHLKPRRALANNNHDSITQFDLSLT |
| TC5031_Tobacco | 0.522 | KLFQLPQQLWLQQPYAGKSRHSQPPHLPYSIPSLRLKIRTFALSDPGASDSTT |
| TC54534_Poplar | 0.574 | EITKATATSTLAAAIHNLSLSSLSSTIRPRPRPYSHSGFSKFPLVSRPTSLTATCSLPNETTTSTTKVAGADTS |
| TC29499_Cotton | 0.591 | TTILHSSLTNPPFTAHLSPSKFPRSFSTPFRLRTSLSFSPSLPPKSFPTNCSVSDDTTSS |
| TC226958_Soybean | 0.542 | EKASLCHRNTPLPLLNRPSNFLQFKHQNSFLKFPNPNLHRLRRLNCSVSDGTVSSSLGATDSS |
| TC94998_Medicago | 0.581 | EKAASSVLKQMNITSRLVLPGSCCCSSFQSSSSSSLHFPFPISVPFKFRSRRSTNSVSDAAFPVPSS |
| TC140119_Barley | 0.581 | AMAMRAAAAFFSPSVSVSPSTSPSPKQRSFFSTRRIRHHRRLRAFPATELTLEELNPSVALLRKT |
| TC237823_Wheat | 0.578 | AMAMRAAAAFFSPSASPSTKQQQTHAVFSTRRGSTRRIHHRRLR |
| TC105593_Sorghum | 0.575 | RAAPAAATGFFSPSSVSPRRFSSATPPASLSTGRCIQRHRLR |
| TC316824_Maize | 0.573 | AMRAATAAATGFFSPSTVPPRRFSSVTPPASLCTARCIQRHRLR |
