# Supplementary material for: Evolutionary Diversification of Plant Shikimate Kinase Gene Duplicates
Source: PLoS Genet. 2008 Dec 5;4(12):e1000292. doi: 10.1371/journal.pgen.1000292 (PMC2593004; doi:10.1371/journal.pgen.1000292)
Supplement: Table S3 — Model M2a posterior probability scores for positively selected sites in the SKL2 family. (0.07 MB DOC) [file pgen.1000292.s007.doc]

| **AtSKL2 Position** | **Prob (ω>1)** | **Mean ω** |
| --- | --- | --- |
| Arg1 | 0.860 | 3.148 |
| Val2 | 0.960 | 3.398 |
| Ser3 | 0.734 | 2.833 |
| Thr4 | 0.573 | 2.432 |
| Ser5 | 0.999 | 3.497 |
| Thr6 | 0.690 | 2.725 |
| Ile7 | 0.999 | 3.497 |
| Gly15 | 0.983 | 3.456 |
| Lys16 | 0.994 | 3.483 |
| Arg23 | 0.992 | 3.478 |
| Lys25 | 0.987 | 3.467 |
| Thr26 | 0.604 | 2.509 |
| Gly27 | 0.980 | 3.449 |
| Glu28 | 0.850 | 3.124 |
| Leu30 | 0.765 | 2.912 |
| Ser31 | 0.629 | 2.571 |
| Pro32 | 0.808 | 3.019 |
| Ala45 | 0.958 | 3.394 |
| Lys47 | 0.612 | 2.529 |
| Arg50 | 0.971 | 3.426 |
| Asn51 | 0.853 | 3.131 |
| Leu53 | 0.995 | 3.487 |
| Leu54 | 0.668 | 2.67 |
| Ile55 | 0.903 | 3.257 |
| Glu59 | 0.607 | 2.516 |
| Asn61 | 0.806 | 3.014 |
| His62 | 0.977 | 3.442 |
| Val88 | 0.582 | 2.454 |
| Gly90 | 0.840 | 3.099 |
| Asp144 | 0.980 | 3.448 |
| Ser151 | 0.762 | 2.903 |
| Ser159 | 0.619 | 2.547 |
| Leu162 | 0.910 | 3.274 |
| Pro166 | 0.690 | 2.723 |
| Ser174 | 0.854 | 3.134 |
| His195 | 0.570 | 2.425 |
| Ala201 | 0.761 | 2.902 |
| Thr221 | 0.931 | 3.326 |
| Lys234 | 0.605 | 2.511 |
| Gln235 | 0.743 | 2.856 |
| Glu236 | 0.866 | 3.163 |
| Arg237 | 0.843 | 3.107 |
| Glu238 | 0.776 | 2.938 |
| Ile239 | 0.837 | 3.092 |
| Gln251 | 0.546 | 2.365 |
| His257 | 0.577 | 2.441 |
| Ser260 | 0.660 | 2.65 |
| Gln271 | 0.814 | 3.033 |
| Ile274 | 0.883 | 3.206 |
| Ser306 | 0.610 | 2.525 |
| Ser307 | 0.803 | 3.006 |
| Asp308 | 0.559 | 2.396 |
| Thr309 | 0.544 | 2.359 |
| His312 | 0.928 | 3.32 |
| Pro313 | 0.603 | 2.506 |
| Gln314 | 0.753 | 2.882 |
