# Supplementary material for: Evolutionary Diversification of Plant Shikimate Kinase Gene Duplicates
Source: PLoS Genet. 2008 Dec 5;4(12):e1000292. doi: 10.1371/journal.pgen.1000292 (PMC2593004; doi:10.1371/journal.pgen.1000292)
Supplement: Table S4 — Primer sequences. (0.03 MB DOC) [file pgen.1000292.s008.doc]

**Primers for recombinant protein expression:**

AtSKL1Δ58 forward (5`- GCGCGCGCTCGAGATGGATCAATCCGCCTCTACTGG -3`)

AtSKL1Δ61 forward (5`-GCGGCGGCTCGAGATGGCCTCTACTGGGATCAG-3`)

AtSKL1Δ64 forward (5`-GCGGCGGCTCGAGATGGGGATCAGTGTTGTTGATTC-3`)

AtSKL1 reverse (5`-GCGCAGATCTCTAGAAAGGTCGAGAAGCTTC-3`)

AtSK2Δ55 forward (5`- GCGGCGGCCCATATGAGATCAGTTTCTGATAAGAAC-3`

AtSK2Δ55 reverse (5`- GCGCGGATCCCTTTAACGCTTCAGTCTCTTTTTC-3`)

AtSK1Δ55 forward (5`- GCGGCGGCCCATATGCAACGAAGAGCAGTTTC-3`

AtSK1Δ55 reverse (5`- GCGCGGATCCCTCTTCTGCTTTTCTCTTGAG-3`)

AtSKL2Δ60 forward (5`- GCGGCGGCCCATATGTTTAATAGCTTCTCATGTAATTG-3`)

AtSKL2Δ60 reverse (5`- GCGCAGATCTTGTAAACTGTGGATGAGGTC-3`)

**Genotyping primers:**

Lba1 (5`-TGGTTCACGTAGTGGGCCATCG-3`)

sk2-1 (5`-ATGCGCTAAGGCTTCTAGAGG-3`) and (5`- TGACAAAAGAAGGGATCATCG-3`)

sk1-1 (5`-GGGTTTTGTGATATTGATGTCCG-3`) and (5`-GAGAGCTTCTTAAGCGCATCGG-3`)

SKL1F (5`-GGAGATCTTCTCTGCGTCTGC-3`)

SKL1R (5`-GAAAGGTCGAGAAGCTTCTTCC-3`)

SKL1E4 (5`-GGAAGCTTTTGGCTGAGG-3`)

SKL1E5 (5`-GCCTTCTCATCAGCTTCCTTC-3`)

**RT-PCR Primers:**

AtSK2 (5`-GGAAGCAGCTACTGTTCAGAGG-3`) and (5`-GACACTCTCACCGAAATGCTC-3`)

AtSK1 (5`-TGACAAAAGAAGGGATCATCG-3`) and (5`-ATGCGCTAAGGCTTCTAGAGG-3`)

AtSKL1 exon 1 (5’-GCTTCTCTAACTTTAACTGGATTCG-3’) and 5’-GCCTTCTCATCAGCTTCCTTC-3’)

AtSKL1 exon 3 (5’-GGAAGCTTTTGGCTGAGG-3’) and (5’-GAAAGGTCGAGAAGCTTCTTCC-3’)
